# Acupuncture versus rehabilitation for post-stroke shoulder-hand syndrome: a systematic review and meta-analysis of randomized controlled trials

**DOI:** 10.3389/fneur.2025.1488767

**Published:** 2025-04-02

**Authors:** Jinyuan Shi, Fuyan Chen, Yang Liu, Mingtong Bian, Xiaowei Sun, Ru Rong, Shuo Liu

**Affiliations:** ^1^Department of Acupuncture, First Teaching Hospital of Tianjin University of Traditional Chinese Medicine, Tianjin, China; ^2^National Clinical Research Center for Chinese Medicine Acupuncture and Moxibustion, Tianjin, China

**Keywords:** acupuncture treatment, post-stroke shoulder-hand syndrome, meta-analysis, rehabilitation, non-pharmacological

## Abstract

**Background:**

Shoulder-hand syndrome (SHS) is one of the common sequelae after stroke, which not only hinders the recovery of patients, but also increases the economic burden of the family. In the absence of effective treatment measures, acupuncture treatment has been widely used in China to treat post-stroke shoulder-hand syndrome, but the details are unclear. Therefore, this review aims to evaluate the true efficacy of acupuncture in patients with SHS.

**Methods:**

We searched eight databases [PubMed, Embase, Web of Science, Cochrane library, China Biomedical Literature Database (CBM), China Science and Technology Journal (VIP) database, the China National Knowledge Infrastructure (CNKI) database, and Wan fang database] from its inception to March 2025, randomized controlled trials (RCTs) of SHS acupuncture treatment combined with rehabilitation (Rehab). Two investigators independently used pre-designed forms to extract valid data from eligible randomized controlled trials. Meta-analysis was implemented through the Rev. Man software (version 5.4). The strength of the evidence obtained was implemented using the GRADE profiler software. Adverse events (AEs) were collected by reading the full text and used to evaluate the safety of acupuncture treatment.

**Results:**

Forty-seven studies, involving 4,129 participants, met the eligibility criteria, and were included in the review. Overall meta-analysis showed that combined acupuncture rehabilitation significantly improved motor function (upper-limb Fugl-Meyer Assessment (FMA): 41 studies, mean difference (MD) 9.50, 95% confidence interval (CI) [8.47, 10.53]) and pain reduction (visual analog score (VAS): 37 studies, MD: −1.49, 95% CI [−1.66, −1.33]). It also improved activities of daily living (ADL) compared to rehabilitation alone (ADL: 17 studies, MD: 11.94, 95% CI [8.26, 13.63]). There was no significant difference in the occurrence of adverse events (AEs) between acupuncture treatment combined with Rehab and Rehab alone (*p* > 0.05). The certainty of the evidence was rated low level because of flaws in the study design and considerable heterogeneity among the included studies.

**Conclusion:**

This review found that acupuncture treatment combined with Rehab treatment may have a positive promoting effect on improving motor function, reducing pain, and improving daily living ability in SHS patients. However, due to the existing methodological quality issues, our findings should be treated with caution. Future high-quality studies are urgently needed to validate our findings.

**Systematic review registration:**

https://www.crd.york.ac.uk/PROSPERO/view/CRD42024536169.

## 1 Introduction

Stroke is the second largest cause of death worldwide and the first leading cause of acquired long-term disability, leading to an annual global economic burden (1). Post-stroke shoulder hand syndrome (PS-SHS), also known as post-stroke complex regional pain syndrome, is experienced by more than 50% of stroke patients (2), it usually occurs between 2 and 3 months of an acute event and usually presents with pain, hyperalgesia, joint swelling, and limited range of motion (3). Post-stroke shoulder hand syndrome is difficult to treat, and its symptoms may persist for a long time, resulting in limited upper limb function or even irreversible permanent apraxia (4, 5). In addition, SHS may hinder the overall recovery, prolong hospitalization, limit the patients’ activities of daily living (ADL), reduce the quality of life, and bring heavy economic burden on the patients and their families (6). Currently, commonly used treatments for post-stroke SHS include drug therapy, soaking in cold water, physical therapy (PT), occupational therapy (OT), psychotherapy, and sympathetic block (7, 8). Although these conventional treatments were initially found to be effective, their adverse effects cannot be ignored. Low-dose oral steroids are effective in improving the SHS after stroke (9), for example, to avoid adverse effects associated with long-term drug use, steroids are only indicated for short-term treatment and are considered a difficult factor for long-term post-stroke SHS (5), early referral to PT, OT, and psychotherapy may prevent the progression of symptoms (10). However, the disadvantages of these approaches, such as high healthcare costs, increased workforce investment, and increased demands for patient collaboration, have also received attention. In addition, the expertise of rehabilitation therapists varies from different regions, which will affect the treatment effect. The incidence of SHS after stroke remains high and is a challenging problem to be addressed. To date, consistently effective and good patient compliance methods are still lacking (11).

As a basic therapy for the prevention and treatment of traditional Chinese medicine (TCM), acupuncture has been used in clinical practice in China for thousands of years (12). According to the site of application, acupuncture can be divided into abdominal acupuncture (AA) and scalp acupuncture (SA). In addition, according to the surgical method, acupuncture can be divided into manual needle (MA), electro-acupuncture (EA), and warm acupuncture (WA) has been proved to have the advantages of low price, good effect and simple operation (13). Moreover, as a non-pharmacological intervention, acupuncture has better efficacy on chronic diseases difficult to treat with traditional treatment methods, such as low back pain and renal disease (14, 15). As a result of these advantages, acupuncture has received continued interest from the general public and health professionals (16). Furthermore, more medical institutions are using acupuncture clinically and show that acupuncture can be used as a prospective therapeutic measure to improve motor function in patients with SHS (6, 17).

In the last few years, three reviews have been published on acupuncture for SHS. The meta-analysis published in 2018 (18) only evaluated the effect of manual acupuncture on the treatment efficacy in patients with SHS, and manual acupuncture represented only one acupuncture form, and the findings were necessarily limited. The meta-analysis published in 2019 (19) included only 13 studies totaling 1,040 patients, an insufficient sample size, and only evaluated the effect of electroacupuncture effects in SHS patients, and the findings were limited. The meta-analysis, published in 2019 (20), selected FMA, VAS and ADL as outcome measures. The results showed that acupuncture treatment had excellent efficacy in relieving the symptoms of SHS. However, the intervention did not involve warm acupuncture. Therefore, we conducted a systematic review of the latest evidence on acupuncture (including warm acupuncture) as an add-on treatment for the clinical treatment of post-stroke SHS. Furthermore, with the widespread use of acupuncture treatment, more research is published in recent years (17, 21). Therefore, the aim of this review was to explore the effect of acupuncture treatment on the relief of clinical symptoms in patients with SHS and to update previous published reviews.

## 2 Materials and methods

### 2.1 Protocol and registration

The detailed protocol of this systematic review and meta-analysis has been registered on the international systematic review registration platform (PROSPERO) with the registration number is CRD42024536169 (22).

### 2.2 Search strategy

Randomized controlled trials (RCTs) on acupuncture treatment SHS from establishment to March 2025 in 8 databases including Web of Science, Embase, PubMed, Cochrane Liberary, Chinese Biomedical Literature Database (CBM), Chinese Science and technology Journal (VIP) database, China National Knowledge Infrastructure (CNKI) database and Wanfang database. Meanwhile, the reference lists of the identified included articles were screened to identify as many relevant articles as possible. No language restrictions were used during the search process. Search terms included “acupuncture treatment,” “electro-acupuncture,” “warm acupuncture,” “shoulder hand syndrome,” “SHS,” “stroke,” “cerebrovascular accident,” “acupuncture,” and “Hao zhen.” The specific search strategies are described in the Appendix.

### 2.3 Literature selection criteria

Two researchers independently screened and checked the titles and abstracts of the literature to be initially included based on the PICOS principles (patient, intervention, control, outcome, and study). The PICOS criteria used for literature screening were detailed as follows: (a) Type of participant: (1) The patient was diagnosed with stroke with no restrictions on age, sex and duration of disease. (2) Their condition was confirmed by magnetic resonance imaging (MRI) or electronic computed tomography (CT) scan. (3) Shoulder hand syndrome is caused by stroke, rather than by other diseases, such as trauma, periarthritis of shoulder, cervical spondylosis; (b) Type of intervention: the experimental group was treated with manual acupuncture (MA), electric acupuncture (EA) or warm acupuncture (WA), with or without the same treatment as the control group. For the control group, rehabilitation (Rehab) must be used and all other types of interventions should be excluded; (c) Type of outcome: Primary outcome measures: (1) motor function: upper limb Fugl-Meyer assessment (FMA) and (2) pain assessment using the visual analog scale (VAS). Secondary outcome measures: (1) Barthel index (BI) or modified Barthel index (MBI) for self-care and activities of daily living; (2) Edema and (3) adverse events. (d) Study type: Randomized Controlled Trial (RCT). The safety of acupuncture treatment was assessed by the severity and number of adverse events (AEs). The language types of these documents are limited to either the Chinese or English language.

Meanwhile, we excluded literature that met the following criteria: (1) Duplication of publications; (2) Studies compared different acupuncture therapies; (3) Full text is not available; (4) Lack of effective outcome measures; (5) Conferences; (6) The types of studies are reviews, animal experiments, conference articles, and case reports.

### 2.4 Data collection process

Two researchers independently used pre-designed forms to retrieve useful information from qualified studies, including publication year, first author, sample size, mean age and method used in experimental groups (e.g., acupuncture modality and acupoint selection), and duration and frequency of treatment. Any inconsistencies in information extraction could be resolved by consulting corresponding author. After data extraction was completed, researchers assessed the safety of acupuncture treatment by collecting adverse event reports from the included articles.

### 2.5 Study quality assessment

The risk of bias in the included randomized controlled trials was assessed using the revised Cochrane Risk of Bias Tool (RoB-2) (23). This evaluation addressed several key aspects: random sequence generation and allocation concealment (both related to selection bias), blinding of participants and personnel (performance bias), blinding of outcome assessment (detection bias), incomplete outcome data (attrition bias), selective reporting (reporting bias), and other potential biases. Each aspect was categorized based on the level of bias risk: low, unclear (indicating some concerns), or high. The findings from this comprehensive bias assessment were then visually represented using Revman 5.4 software, offering a clear graphical depiction of the potential biases within these trials.

### 2.6 GRADE assessment

Two researchers (JY-S and Y-L) assessed the quality of evidence for each outcome indicator by using the Grading of Recommendations, Assessment, Development, and Evaluation (GRADE) system (24). When disagreements arose, they could be resolved through consultation or by consulting a third researcher. The quality of evidence for each outcome is displayed in the form of a GRADE evidence profile to determine the certainty of all pooled outcomes. The GRADE system includes five downgrading factors and three escalating factors, five downgrading factors consisting of risk of bias, inconsistency, indirectness, uncertainty, and publication bias, and three escalating factors consisting of larger effect values, dose effect relationships, and negative bias. The quality of evidence for each outcome was assessed by the eight factors mentioned above, which ultimately resulted in a high, moderate, low, or very low evidence level.

### 2.7 Statistical analysis

After data extraction was completed, Rev. Man 5.4 software (Cochrane Collaboration, Oxford, United Kingdom) was selected for statistical analysis. If the outcome indicator was a continuous variable, mean difference (MD) or standardized mean difference (SMD) and 95% confidence interval (CI) were selected for calculation. If the outcome indicator was a dichotomous variable, the risk ratio (RR) and 95% CI were selected for calculation. Heterogeneity between included studies was assessed using *Q*-tests (*p*-values) and the *I*^2^ statistic, and effect models were selected accordingly. If *p* < 0.1 and *I*^2^ > 50%, statistically significant heterogeneity among the included studies was detected and a random effects model was selected to calculate the effect size. Conversely, if *p* ≥ 0.1 and *I*^2^ ≤ 50%, the heterogeneity among the included studies was regarded as tolerable and we selected the fixed effects model to merge the data. This meta-analysis assessed the significance of the pooled results by *Z*-test, with *p* < 0.05 being a statistically significant difference.

We categorized the included studies according to different acupuncture types and treatment duration, which were MA, EA, WA, 0–4 weeks, and > 4 weeks. Subgroup analysis was attempted to account for possible heterogeneity under the stratification factors of different acupuncture types and treatment cycles. Sensitivity analyses were used to validate the robustness of the meta-analysis results and to explore potential sources of heterogeneity by excluding each individual study in the original analysis. For FMA, VAS, ADL, we used funnel plots and Egger’s tests to evaluate publication bias in the included studies.

## 3 Results

### 3.1 Description of the studies

**Figure 1:**
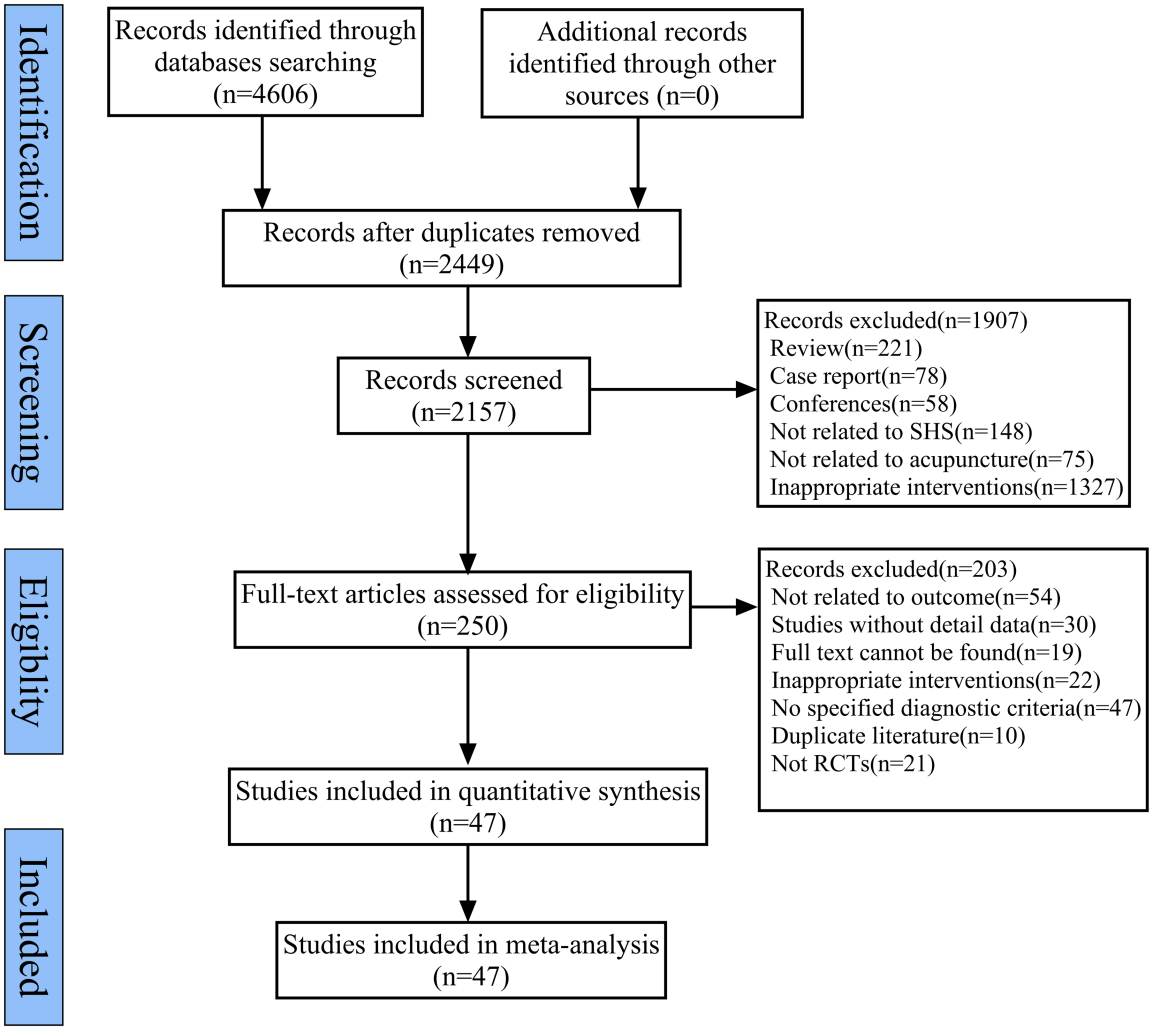
Flow chat of the literature screening process.

Using a pre-defined search strategy, we initially retrieved 4,606 relevant records from eight databases. A total of 4,356 duplicate and irrelevant studies were excluded by screening titles and abstracts. Subsequently, the full texts of the remaining studies were reviewed, resulting in the exclusion of 203 records. Finally, 47 studies were included in the qualitative analysis (25–71). All included studies were conducted in China, consisting of 46 RCTs published in Chinese and 1 RCT in English. These studies were published between 2008 and 2025. Figure 1 shows the flow chart of the literature screening process for the systematic review and meta-analysis.

### 3.2 Description of participants

A total of 4,129 participants were included, with 2,068 in the experimental group and 2,061 in the control group. The sample size per study ranged from 40 to 178. The proportion of males was higher than that of females. The mean age of patients was similar in both groups. Among the studies, 29 reported SHS staging, including 21 studies on stage I (25, 26, 29, 31, 33, 35, 38, 40, 43, 44, 46, 49–52, 59, 60, 65, 66, 69, 70). Two studies (65, 70) reported participant dropouts (3 and 5 cases, respectively) with corresponding reasons provided. Details of the 47 included RCTs are summarized in Table 1.

**Table 1:** Characteristics of included studies.

References	Sample size (EG/CG)	Gender (M/W)	Mean age (EG/CG)	Type of stroke	Disease duration (EG/CG)	SHS severity	Method of intervention	Comparison	Outcome	Loss situation
Chen et al. (25)	48/46	43/51	58.32 ± 11.56/60.12 ± 12.48	Ischemic stroke or hemorrhage	34.91 ± 14.66/35.41 ± 16.90 d	Stage I	MA	Rehab	FMA VAS BI	/
Chen and Guan (26)	60/60	68/52	62.18 ± 4.23/62.25 ± 4.11	Ischemic stroke	4.45 ± 1.20/4.24 ± 1.14 m	Stage I	MA	Rehab	FMA VAS Edema	/
Dong et al. (27)	45/45	53/37	60.25 ± 5.27/61.95 ± 4.95	Ischemic stroke or hemorrhage	8.25 ± 4.19/8.95 ± 4.55 m	Stage I, II, III	MA	Rehab	FMA VAS	/
Dou (28)	35/35	44/26	52.3 ± 8.9/52.1 ± 9.2	Ischemic stroke or hemorrhage	21.4 ± 7.6/21.8 ± 7.5 d	/	MA	Rehab	MBI	/
Fang and Cao (29)	45/45	66/25	50 ± 2.7/58 ± 4.5	Ischemic stroke or hemorrhage	36 ± 14.8/35 ± 16.5 d	Stage I	MA	Rehab	FMA	/
Feng (30)	30/31	49/11	63.6 ± 8.4/62.9 ± 7.2	Ischemic stroke or hemorrhage	48.6 ± 5.17/50.7 ± 4.87 d	/	EA	Rehab	MBI	/
Hou (31)	30/30	33/27	62.3 ± 5.6/63.1 ± 6.3	Ischemic stroke or hemorrhage	29.8 ± 6.2/31.5 ± 6.9 d	Stage I	EA	Rehab	FMA VAS	/
Huang et al. (32)	80/80	90/70	59.63 ± 5.52/59.48 ± 6.13	Ischemic stroke or hemorrhage	1.5–7/1–6 m	/	WA	Rehab	FMA VAS	/
Jia et al. (33)	28/24	31/21	60.5 ± 5.3/61.3 ± 5.9	Ischemic stroke or hemorrhage	28.5 ± 5.7/31.0 ± 7.3 d	Stage I	EA	Rehab	FMA VAS	/
Lei et al. (34)	31/31	38/24	48.3 ± 8.1/49.2 ± 7.5	Ischemic stroke or hemorrhage	32.3 ± 6.1/33.4 ± 5.5 d	/	EA	Rehab	FMA VAS	/
Li and Tu (35)	45/45	50/40	51.18 ± 7.60/50.69 ± 6.99	Ischemic stroke or hemorrhage	4.29 ± 1.87/3.39 ± 1.64 d	Stage I	EA	Rehab	FMA VAS MBI	/
Li and Li (36)	35/35	48/22	55.5 ± 13.0/55.3 ± 13.2	Ischemic stroke or hemorrhage	47.5 ± 11.6/47.2 ± 11.9 d	Stage I, II	EA	Rehab	FMA VAS	/
Liang and Lui (37)	42/42	58/26	56.5 ± 3.2/55.8 ± 3.6	Ischemic stroke or hemorrhage	10d-3 m/15d-3 m	Stage I, II	EA	Rehab	FMA VAS	/
Lin (38)	89/89	111/67	60.76 ± 8.17/60.33 ± 8.26	Ischemic stroke or hemorrhage	36.82 ± 10.29/36.37 ± 10.48 d	Stage I	MA	Rehab	FMA VAS MBI	/
Liu (39)	40/40	41/39	57.05 ± 10.74/61.85 ± 11.30	Ischemic stroke or hemorrhage	36.25 ± 8.42/37.25 ± 8.23 d	/	MA	Rehab	FMA VAS	/
Liu (40)	38/38	44/32	67.1 ± 3.4/68.3 ± 3.1	Ischemic stroke or hemorrhage	30.8 ± 8.5/31.2 ± 7.8 d	Stage I	EA	Rehab	FMA VAS	/
Liu (42)	46/46	55/37	62.51 ± 10.39/62.79 ± 10.53	Ischemic stroke or hemorrhage	32.09 ± 5.28/31.84 ± 5.49 d	/	WA	Rehab	FMA Edema	/
Liu and Jiao (41)	49/49	53/46	62.18 ± 11.61/63.21 ± 10.95	Ischemic stroke or hemorrhage	48.18 ± 13.61/47.18 ± 14.12 d	/	MA	Rehab	FMA VAS	/
Lu et al. (43)	40/40	47/33	62.2 ± 8.6/61.9 ± 8.4	Ischemic stroke or hemorrhage	29.4 ± 8.4/28.4 ± 8 d	Stage I	MA	Rehab	FMA VAS	/
Nie and Zhao (44)	20/20	27/13	68 ± 6/57 ± 6	Ischemic stroke or hemorrhage	18.12 ± 4.76/16.78 ± 4.23 d	Stage I	WA	Rehab	FMA VAS	/
Niu (45)	54/54	65/43	62.4 ± 9.6/62.9 ± 9.4	Ischemic stroke or hemorrhage	3.2 ± 0.9/3.1 ± 0.6 m	/	MA	Rehab	FMA VAS MBI	/
Shao et al. (46)	30/30	33/27	54.07 ± 6.36/56.33 ± 5.43	Ischemic stroke or hemorrhage	7.13 ± 4.79/6.98 ± 3.47 m	Stage I	MA	Rehab	FMA VAS	/
Shao et al. (47)	32/32	35/29	58.17 ± 7.01/59.24 ± 6.54	Ischemic stroke or hemorrhage	35.23 ± 2.31/34.83 ± 3.34 d	/	MA	Rehab	VAS BI	/
Shen (48)	30/30	31/29	61.2 ± 6.7/61.1 ± 6.6	Ischemic stroke or hemorrhage	27.2 ± 7.8/27.1 ± 7.7 d	/	MA	Rehab	FMA	/
Song and Xie (49)	98/98	112/84	62 ± 5/61 ± 6	Ischemic stroke	39.43 ± 3.85/38.57 ± 4.61 d	Stage I	EA	Rehab	FMA VAS Edema	/
Sun et al. (50)	30/30	39/21	60.4 ± 8.7/59.2 ± 8.5	Ischemic stroke	45.2 ± 9.6/46.7 ± 9.1 d	stage I	MA	Rehab	FMA VAS	/
Tang et al. (52)	30/30	39/21	57.92 ± 8.16/59.30 ± 8.47	Ischemic stroke	48.54 ± 9.62/49.12 ± 9.37 d	Stage I	MA	Rehab	FMA VAS	/
Wang et al. (53)	71/71	73/69	65.0 ± 4.3/64.2 ± 3.6	Ischemic stroke or hemorrhage	66.8 ± 11.4/67.4 ± 10.5 d	stage I, II, III	MA	Rehab	FMA MBI	/
Wang et al. (54)	50/50	57/43	61.56 ± 6.04/61.27 ± 5.87	Ischemic stroke or hemorrhage	1.76 ± 0.51/1.74 ± 0.48 m	Stage I, II	MA	Rehab	FMA VAS	/
Wang and Li (55)	30/30	31/29	57.50 ± 6.72/57.38 ± 6.64	Ischemic stroke or hemorrhage	4.53 ± 0.82/4.61 ± 1.07 m	/	MA	Rehab	FMA VAS	/
Wen and Zhang (56)	30/30	37/23	55.46 ± 6.47/54.32 ± 6.58	Ischemic stroke or hemorrhage	5.76 ± 1.85/5.48 ± 1.76 m	Stage I, II	MA	Rehab	FMA	/
Xie and Li (58)	40/40	49/21	50 ± 11/51 ± 10	Ischemic stroke or hemorrhage	14d-2 m/15d-2 m	/	EA	Rehab	FMA VAS	/
Xie et al. (59)	53/53	52/54	59.64 ± 6.75/60.01 ± 6.43	Ischemic stroke or hemorrhage	29.23 ± 5.46/30.18 ± 6.19 d	Stage I	WA	Rehab	FMA VAS	/
Xu et al. (60)	42/40	43/39	64.36 ± 6.32/62.73 ± 7.26	Ischemic stroke	33.45 ± 6.23/27.14 ± 7.53 d	/	MA	Rehab	FMA VAS	/
Xu et al. (61)	40/40	49/31	59.6 ± 8.7/60.3 ± 9.1	Ischemic stroke	47.2 ± 9.6/48.6 ± 9.3 d	Stage I	MA	Rehab	FMA VAS Edema	/
Yang et al. (62)	39/39	49/29	54.37 ± 13.24/55.84 ± 12.76	Ischemic stroke or hemorrhage	3.58 ± 2.13/3.85 ± 2.33 m	Stage I, II	WA	Rehab	FMA VAS	/
Yang (71)	43/43	49/37	60.47 ± 4.02/61.03 ± 4.87	Ischemic stroke or hemorrhage	20.51 ± 1.41/21.81 ± 1.50 d	/	WA	Rehab	FMA VAS MBI	/
Yang (63)	50/50	53/47	52.11 ± 9.23/52.31 ± 8.91	Ischemic stroke or hemorrhage	21.82 ± 7.52/21.41 ± 7.61 d	/	MA	Rehab	VAS BI	/
You (64)	40/40	55/25	60.87 ± 11.34/61.23 ± 10.92	Ischemic stroke or hemorrhage	33.45 ± 18.11/34.23 ± 16.25 d	/	EA	Rehab	FMA VAS MBI	/
Zheng et al. (65)	39/38	46/31	58.54 ± 6.05/59.62 ± 5.71	Ischemic stroke or hemorrhage	41.72 ± 5.04/42.48 ± 6.64 d	Stage I	MA	Rehab	FMA VAS BI	3
Zheng et al. (67)	89/89	107/71	54.25 ± 3.15/53.35 ± 3.30	Ischemic stroke or hemorrhage	41.43 ± 8.01/42.03 ± 7.38 d	/	MA	Rehab	FMA VAS	/
Zheng (68)	43/43	47/39	52.41 ± 1.05/52.36 ± 1.28	Ischemic stroke or hemorrhage	15.48 ± 2.06/15.36 ± 2.31 d	/	WA	Rehab	FMA VAS MBI	/
Zhong et al. (68)	30/30	30/29	62.5 ± 7.4/62.3 ± 8.9	Ischemic stroke or hemorrhage	28.4 ± 10.3/29.2 ± 9.8 d	Stage I	MA	Rehab	FMA VAS	/
Zhu and Zhou (70)	30/30	27/33	59.30 ± 7.77/58.53 ± 7.36	Ischemic stroke or hemorrhage	35.33 ± 9.06/33.47 ± 8.74 d	Stage I	MA	Rehab	FMA VAS	5
Wu et al. (57)	58/58	69/47	68.28 ± 4.72/68.52 ± 4.87	Ischemic stroke or hemorrhage	29.11 ± 6.13/28.58 ± 5.27	Stage I, II	MA	Rehab	FMA VAS MBI	/
Zhang et al. (66)	40/40	51/29	62.35 ± 8.51/63.82 ± 8.74	Ischemic stroke	35.19 ± 10.67/36.73 ± 12.55	Stage I	EA	Rehab	FMA	/
Sun and Tu (51)	30/30	33/27	63.58 ± 1.10/62.35 ± 1.25	Ischemic stroke	(3.12 ± 0.43/3.17 ± 0.45)	Stage I	MA	Rehab	FMA VAS BI	/

EG, Experimental group; CG, Control group; MA, manual acupuncture; EA, electroacupuncture; WA, warm acupuncture; Rehab, rehabilitation; FMA, Fugl-Meyer Assessment (upper limb); VAS, visual analog scale; BI, Barthel Index; MBI, Modified Barthel Index; SHS, shoulder-hand sydrome.

### 3.3 Description of interventions

In the included studies, manual acupuncture was the most frequently used intervention (59.57%), followed by electroacupuncture (25.53%) and warm acupuncture (14.89%). All included studies used Rehab as a control measure. The retention time ranged from 15 to 40 min, with 30 min being the most common duration (*n* = 35). The treatment frequency ranged from 3 to 7 times per week, with 5 times weekly being the most common pattern (*n* = 13). The treatment duration varied widely across studies, ranging from 2 to 8 weeks, where a 4-week treatment period was the most common (*n* = 21). Through analysis of acupoint selection patterns, we found that acupuncture points for SHS were primarily located in the shoulder and arm regions. The most frequently used acupoints were Wai-guan (SJ5) (63.83%), Jian-yu (LI15) (63.83%), He-gu (LI4) (61.70%), Qu-chi (LI11) (61.07%), Jian-liao (SJ14) (38.30%), Shou-san-li (LI10) (38.30%) and Jian-zhen (SI9) (31.91%). Table 2 details the intervention characteristics of the included studies.

**Table 2:** Details of interventions in included studies.

References	Method of intervention	Acupiont selection	Frequency	Retained	Course	Adverse event
Chen et al. (25)	MA	Jian-yu (LI15), Jian-liao (TE14), Jian-zhen (SI9), Jian-qian, Qu-chi (LI11), Wai-guan (TE5), Shou-san-li (LI10), Ji-quan (HT1), Chi-ze (LU5), Nei-guan (PC5)	Once/d, 6 times/week	30 min	4 weeks	/
Chen Guan (26)	MA	Nei-guan (PC5), Shou-san-li (LI10), Wai-guan (TE5), Qu-chi (LI11), He-gu (LI4), Jian-yu (LI15)	Once/d	30 min	4 weeks	None
Dong et al. (27)	MA	Posterior parietotemporal oblique line, Top side 2 lines, Jian-yu (LI15), Jian-liao (TE14), Bi-nao (LI14), Hou-xi (SI3), Hou-xi (SI3), Jian-zhen (SI9), Jian-jing (GB21), Wai-guan (TE5), Zhong-zhu (TE3), Tian-zong (SI11), Yang-xi (LI5)	Once/d	15 min	4 weeks	/
Dou (28)	MA	He-gu (LI4), Shou-san-li (LI10), Wai-guan (TE5), Nei-guan (PC5), Ji-quan (HT1), Jian-yu (LI15), Qu-chi (LI11), Chi-ze (LU5), Jian-zhen (SI9), A-shi, Jian-qian,	Once/d, 4 times/week	30 min	5 weeks	/
Fang and Cao (29)	MA	2/5 middle parietotemporal oblique line, Top side 2 lines	Once/d, 6 times/week	30 min	4 weeks	/
Feng (30)	EA	A-shi, Jian-yu (LI15), Tian-zong (SI11), Shou-san-li (LI10), Qu-chi (LI11), Nei-guan (PC5), He-gu (LI4), Wai-guan (TE5)	5 times/week	20 min	5 weeks	/
Hou (31)	EA	Wai-guan (TE5), Qu-chi (LI11), He-gu (LI4), Bai-hui (GV20), Shen-ting (GV24), Yin-tang (GV29)	5 times/week	30 min	4 weeks	/
Huang et al. (32)	WA	Tian-zong (SI11), Jian-yu (LI15), Jian-zhen (SI9), Hou-xi (SI3), Zhi-zheng (SI7), Xiao-hai (SI8), Shao-ze (SI1), A-shi	Once/d	30-40 min	4 weeks	/
Jia et al. (33)	EA	Jian-yu (LI15), Jian-liao (TE14), Qu-chi (LI11), Wai-guan (TE5), He-gu (LI4), Bai-hui (GV20), Yin-tang (GV29), Shen-ting (GV24)	Once/d, 5 times/week	30 min	4 weeks	/
Lei et al. (34)	EA	Jian-liao (TE14), Jian-yu (LI15), Jian-jing (GB21), Jian-zhen (SI9), He-gu (LI4), Wai-guan (TE5), Qu-chi (LI11), Zhong-zhu (TE3), Tian-zong (SI11), Bi-nao (LI4), Ye-men (TE2), Shi-xuan (EX-UE11)	Once/d	30 min	20d	/
Li and Tu (35)	EA	Jian-liao (TE14), Shou-san-li (LI10), He-gu (LI4), Wai-guan (TE5), Nei-guan (PC6), Qu-chi (LI11), Jian-yu (LI15), A-shi	Once/d, 5 times/week	30 min	6 weeks	/
Li and Li (36)	EA	Jian-yu (LI15), He-gu (LI4), Wai-guan (TE5), Shou-san-li (LI10), Qu-chi (LI11)	Once/d	30 min	21d	Subcutaneous hemorrhage
Liang and Lui (37)	EA	Jian-liao (TE14), Jian-zhen (SI9), Jian-yu (LI15), Jian-jing (GB21), Qu-chi (LI11), He-gu (LI4), Wai-guan (TE5), Tian-zong (SI11), Bi-nao (LI4), Hou-xi (SI3), Lao-gong (PC8)	Once/d	30 min	30d	/
Lin (38)	MA	Ba-xie (EX-UE10), Wai-lao-gong (EX-UE8)	Once/d, 5 times/week	20 min	4 weeks	/
Liu (39)	MA	Jian-tong	Once/d	30 min	20d	/
Liu (40)	EA	He-gu (LI4), Qu-chi (LI11), Bai-hui (GV20), Yin-tang (GV29), Shen-ting (GV24), Wai-guan (TE5)	Once/d, 5 times/week	30 min	4 weeks	/
Liu (42)	WA	Jian-yu (LI15), Qu-chi (LI11), Wai-guan (TE5), Shou-san-li (LI10), He-gu (LI4)	Once/d, 5 times/week	30 min	4 weeks	/
Liu and Jiao (41)	MA	He-gu (LI4), Wai-guan (TE5), Jian-yu (LI15), Qu-chi (LI11), Hou-xi (SI3), Zhong-zhu (TE13)	Once/d, 6 times/week	20 min	4 weeks	/
Lu et al. (43)	MA	Shui-gou (GV26), Nei-guan (PC6)	Once/d	30 min	1 m	/
Nie and Zhao (44)	WA	Wai-guan (TE5), Yang-chi (TE4), Qu-chi (LI11), He-gu (LI4), Jian-yu (LI15), Wan-gu (SI4), Jian-jing (GB21)	5 times/week	30 min	2 weeks	None
Niu (45)	MA	Qu-chi (LI11), He-gu (LI4), Jian-yu (LI15), Wai-guan (TE5), Bai-hui (GV20), Shen-ting (GV24), Jian-qian	Once/d	30 min	4 weeks	/
Shao et al. (46)	MA	Jian-zhen (SI9), Jian-liao (TE14), Jian-qian, Shou-san-li (LI10), He-gu (LI4), Tian-zong (SI11), He-gu (LI4), Wai-guan (TE5), Chi-ze (LU5), Qu-ze (PC3)	Once/d, 6 times/week	30 min	8 weeks	/
Shao et al. (47)	MA	Xuan-zhong (GB39), Zu-san-li (ST39), Qu-chi (LI11), Jian-yu (LI15), Shou-san-li (LI10), Wai-guan (TE5), San-yin-jiao (SP6), Jian-liao (TE14), Tai-chong (LR3), He-gu (LI4), Jian-qian	Once/d	30 min	4 weeks	/
Shen (48)	MA	Ren-ying (ST9), Chi-ze (LU5), Nei-guan (PC6), Ji-quan (HT1), Yang-xi (LI5)	Once/d	30 min	40 d	/
Song and Xie (49)	EA	Nei-guan (PC5), Jian-yu (LI15), Wai-guan (TE5), Jian-liao (TE14), Zu-san-li (ST39), San-yin-jiao (SP6), Qu-chi (LI11), Xuan-zhong (GB39), Shou-san-li (LI10)	Once/d, 7 times/week	30 min	4 weeks	/
Sun et al. (50)	MA	Jianqian (Extra), Yuji (LU10), Jianyu (LI15), Hegu (LI 4), Jianliao (TE 14), Zhongzhu (TE 3), Naoshu (SI 10), Houxi (SI 3)	6 times/week	20 min	3 weeks	/
Tang et al. (52)	MA	Tian-zong (SI11), Jian-liao (TE14), Zhong-zhu (TE3), Jian-qian, Bi-nao (LI4), Hou-xi (SI3)	Once/d, 6 times/week	30 min	28 d	/
Wang et al. (53)	MA	Jian-liao (TE14), Jian-yu (LI15), Jian-jing (GB21), Jian-zhen (SI9), He-gu (LI4), Wai-guan (TE5), Qu-chi (LI11), Zhong-zhu (TE3), Yang-lao (SI6), Zhong-zhu (TE13), Zhong-zhu (TE13), Qi-hai (CV6), Zu-san-li (ST39), Tian-ding (LI17)	Once/d	15 min	21 d	/
Wang et al. (54)	MA	Yang-lao (SI6)	Once/d	20 min	8 weeks	Pain, Red and swollen, Pruritus
Wang and Li (55)	MA	Jian-yu (LI15), He-gu (LI4), Wai-guan (TE5), Shou-san-li (LI10), Qu-chi (LI11), Jian-zhen (SI9), Jian-jing (GB21), A-shi	Once/d, 5 times/week	30 min	4 weeks	/
Wen and Zhang (56)	MA	parietal line mid, Top slope 1 line, Top slope 2 lines, Top next to the line, Jian-yu (LI15), Jian-liao (TE14), Bi-nao (LI14), Qu-chi (LI11), Wai-guan (TE5), Zu-san-li (ST39), Jian-qian, Si-qiang	Twice/d	30 min	30 d	/
Xie and Li (58)	EA	Shou-san-li (LI10), Qu-chi (LI11), Qing-leng-yuan (TE11), Nao-hui (TE13), Jian-yu (LI15), He-gu (LI4), Yang-xi (LI5)	3 times/week	30 min	4 weeks	/
Xie et al. (59)	WA	Qu-chi (LI11), He-gu (LI4), Wai-guan (TE5), Nei-guan (PC6), Shou-san-li (LI10), Ji-quan (HT1), Chi-ze (LU5)	Once/d, 5 times/week	3 column	3 m	/
Xu et al. (60)	MA	Jian-san-zhen, Ji-quan (HT1), Chi-ze (LU5), Nei-guan (PC6)	Once/d, 6 times/week	30 min	5 weeks	None
Xu et al. (61)	MA	Jian-yu (LI15), Bi-nao (LI14), Jian-qian, Jian-hou, Ji-quan (HT1), Zhong-zhu (TE13), Hou-xi (SI3)	Once/d	40 min	14 d	/
Yang et al. (62)	WA	Gan-shu (BL8), Ge-shu (BL17), Pi-shu (BL20), Shen-shu (BL23), San-yin-jiao (SP6), Shen-ting (GV24), Yin-tang (GV29), Xuan-lu (GB5), Shuai-gu (GB8), Nao-kong (GB19), Qiang-jian (GV18), Jian-zhen (SI9), Jian-liao (TE14), Shou-san-li (LI10), Xi-men (PC4)	Once/d	20 min	30 d	/
Yang (71)	WA	Qu-chi (LI11), He-gu (LI4), Wai-guan (TE5), Jian-yu (LI15), Bi-nao (LI14), Chi-ze (LU5), Zu-san-li (ST39), Tai-yuan (LU9), Jian-qian, Jian-hou	Once/d, 5 times/week	20 min	4 weeks	/
Yang (63)	MA	Zu-san-li (ST39), Xuan-zhong (GB39), Jian-yu (LI15), He-gu (LI4), Hou-xi (SI3), Qu-chi (LI11), Tai-chong (LR3), Huan-tiao (GB30), Wai-guan (TE5), Yang-ling-quan (GB34), Bi-guan (ST31)	Once/d	30 min	20 d	/
You (64)	EA	He-gu (LI4), Wai-guan (TE5), Shou-san-li (LI10), Jian-yu (LI15), Jian-liao (TE14), Bi-nao (LI14), Jian-zhen (SI9), Tian-zong (SI11), Ba-xie (EX-UE8)	Once/d, 6 times/week	30 min	3 weeks	/
Zheng et al. (65)	MA	Jian-liao (TE14), Jian-yu (LI15), Jian-zhen (SI9), He-gu (LI4), Wai-guan (TE5), Qu-chi (LI11), Hou-xi (SI3), Shou-san-li (LI10)	Once/d, 5 times/week	30 min	4 weeks	/
Zheng et al. (67)	MA	Jian-yu (LI15), Jian-liao (TE14), Jian-zhen (SI9), Wai-guan (TE5), He-gu (LI4), Shou-san-li (LI10), Qu-chi (LI11)	Once/d	30 min	1 m	/
Zheng (68)	WA	He-gu (LI4), Hou-xi (SI3), Yu-ji (LU10), Zhong-zhu (TE13), Jian-zhen (SI9), Yang-chi (TE4)	Once/d	30 min	30 d	/
Zhong et al. (68)	MA	Pian-tan, Jian-tong, Tou-sheng-ti, Jian-san-zhen, A-shi, Ji-quan (HT1), Chi-ze (LU5), Nei-guan (PC6)	Once/d	30 min	30 d	/
Zhu and Zhou (70)	MA	Jian-yu (LI15), Jian-liao (TE14), Wai-guan (TE5), He-gu (LI4), Qu-chi (LI11), Hou-xi (SI3)	Once/d	30 min	4 weeks	/
Wu et al. (57)	MA	Bai-hui (GV20), Shui-gou (GV26), Zhong-chong (PC9), Qu-chi (LI11), He-gu (LI4), Jian-yu (LI15), Xuan-zhong (GB39), Yang-ling-quan (GB34), Huan-tiao (GB30), Feng-shi (GB31)	Once/d, 5 times/week	30 min	4 weeks	/
Zhang et al. (66)	EA	Jian-yu (LI15), Qu-chi (LI11), He-gu (LI4), Jian-liao (TE14), Wai-guan (TE5), Jian-zhen (SI9), Shou-san-li (LI10), Tian-zong (SI11), Nei-guan (PC5)	Once/d, 5 times/week	30 min	30 d	
Sun and Tu (51)	MA	Nei-guan (PC5), Shui-gou (GV26), Ji-quan (HT1), Chi-ze (LU5)	Once/d	30 min	4 weeks	

MA, manual acupuncture; EA, electroacupuncture; WA, warm acupuncture; /, not mention.

### 3.4 Methodological quality

**Figure 2:**
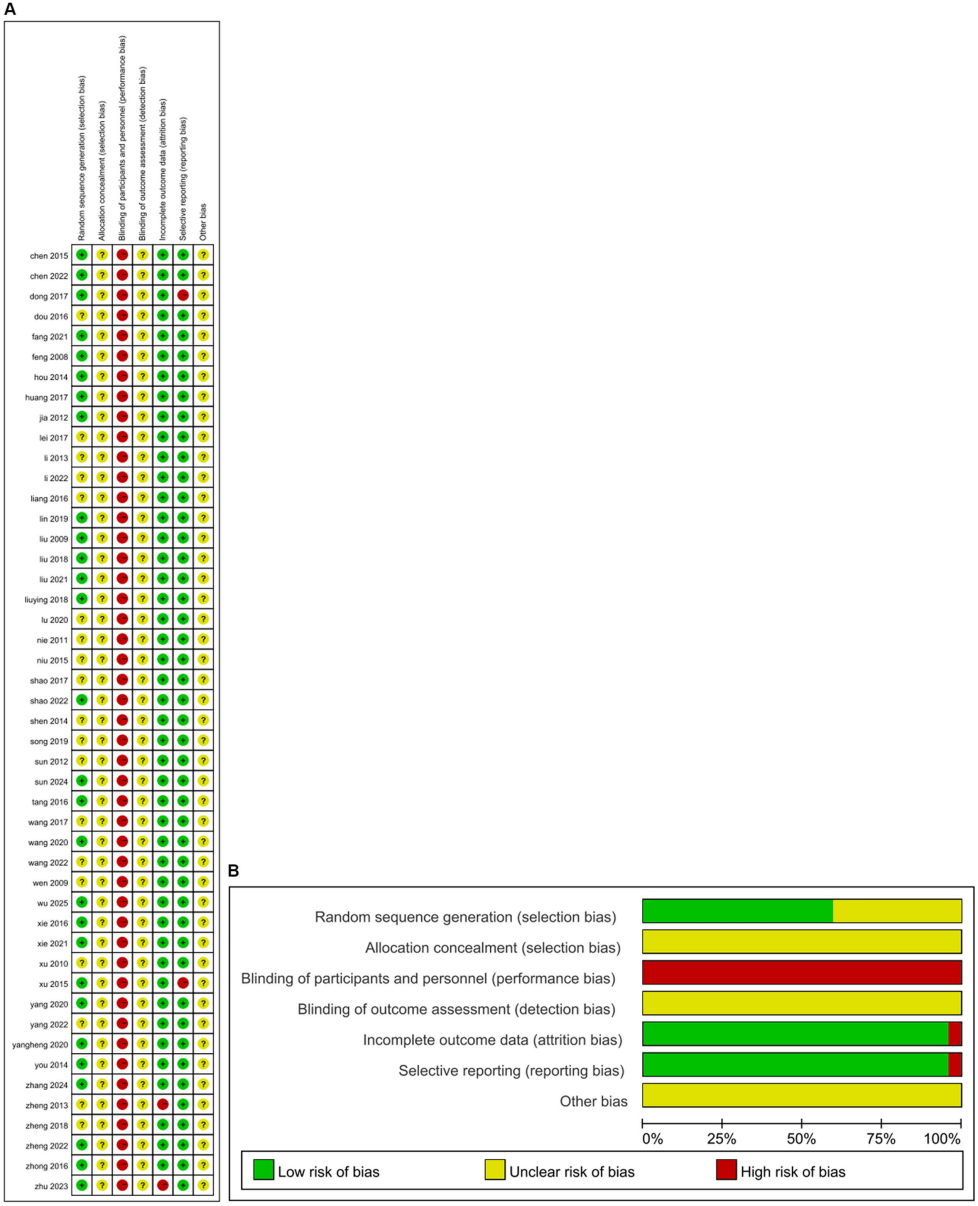
The figure represents the risk of bias assessment for the studies selected in the meta-analysis. **(A)** ROB graph. **(B)** ROB summary.

The results of the methodological assessment are shown in Figure 2. Twenty-eight studies (25–27, 29–33, 38–42, 47, 51, 52, 54, 57–60, 62–64, 66, 68–70), were rated as low risk due to the use of random number tables or computer-generated randomization for sequence generation, while the remaining 19 studies (28, 34–37, 43–46, 48–50, 53, 55, 56, 61, 65, 67, 71) were classified as having unclear risk of bias due to insufficient information. None of the 47 studies described the allocation concealment process in sufficient detail, resulting in an unclear risk of bias judgment. Blinding of participants or personnel could not be implemented in any of the 47 studies because of significant differences in acupuncture treatment protocols between the intervention and control groups. The two studies (65, 70) were rated as high risk because they did not conduct appropriate intention-to-treat analysis in the context of the dropout situation. Forty-five studies were categorized as low risk of selective reporting bias because all pre-specified endpoints were reported, while two studies (27, 61) were rated as high risk of selective reporting bias due to incomplete endpoint reporting. For the remaining 47 studies, insufficient data were available to assess other potential sources of bias.

### 3.5 Meta-analysis

#### 3.5.1 FMA

**Figure 3:**
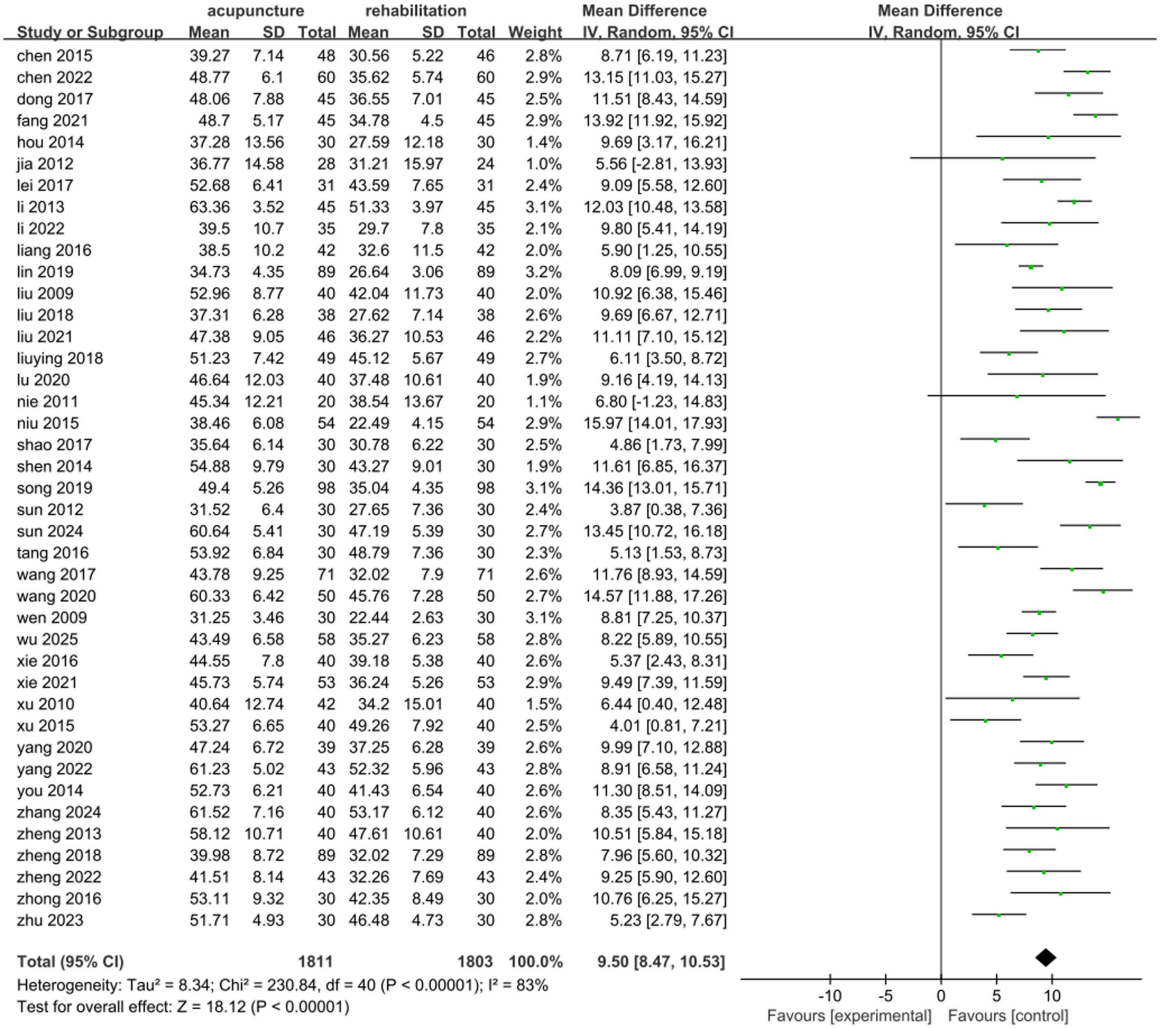
Forest plot of acupuncture treatment combined with Rehab vs. Rehab on FMA.

Forty-one studies (25–27, 29, 31, 33–46, 48–62, 64–71) involving 3,614 patients reported increased FMA scores with acupuncture combined with Rehab compared to Rehab alone. These studies showed significant statistical heterogeneity in FMA outcomes (*p* < 0.00001, *I*^2^ = 83%). Using a random-effects model, the pooled MD was 9.50 (95% CI: 8.47, 10.53). The results demonstrated a statistically significant difference between combined therapy and Rehab alone in the overall effect size (*Z* = 18.12, *p* < 0.00001; see Figure 3).

**Figure 4:**
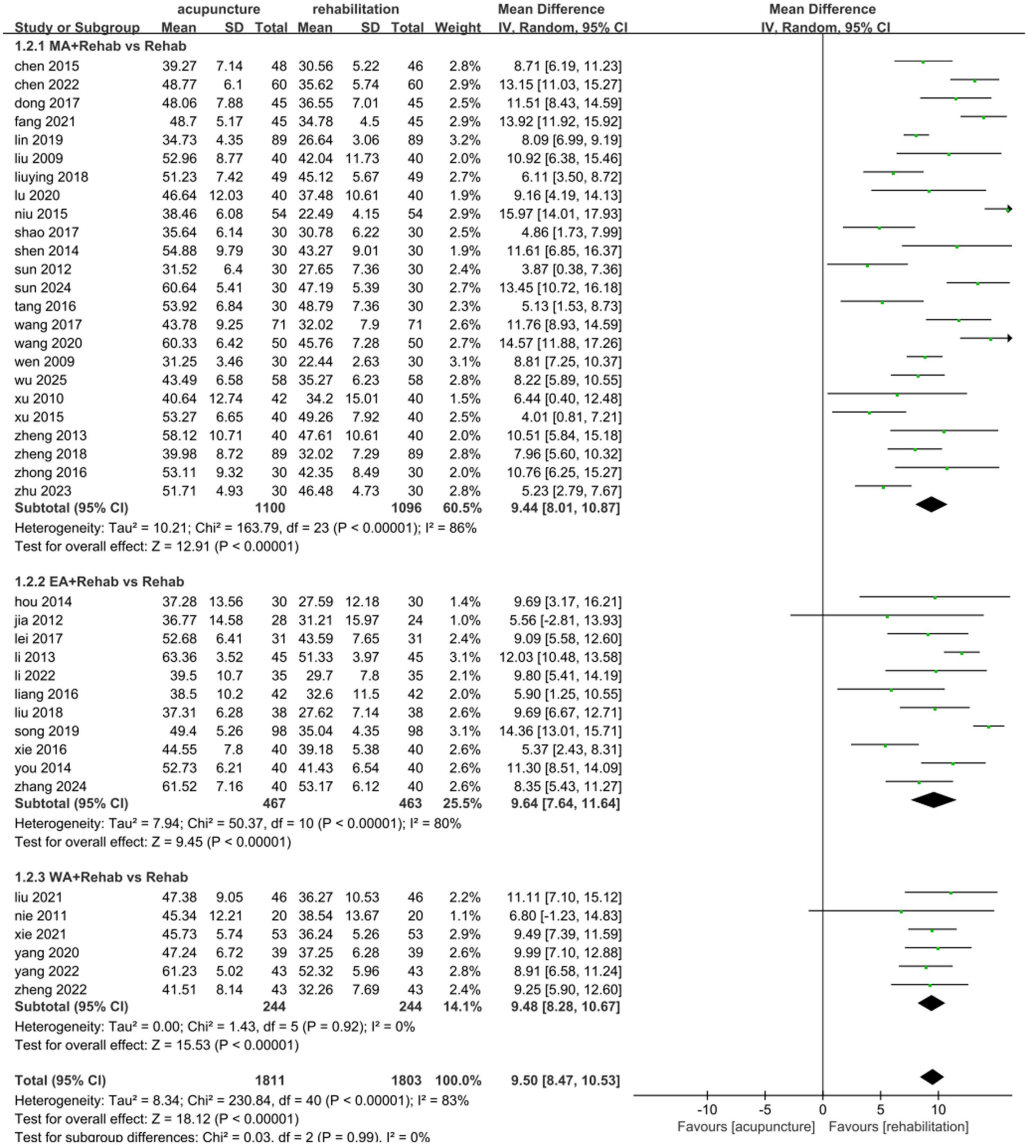
Forest plot of the intervention subgroup of acupuncture treatment combined with Rehab vs. Rehab on FMA.

**Figure 5:**
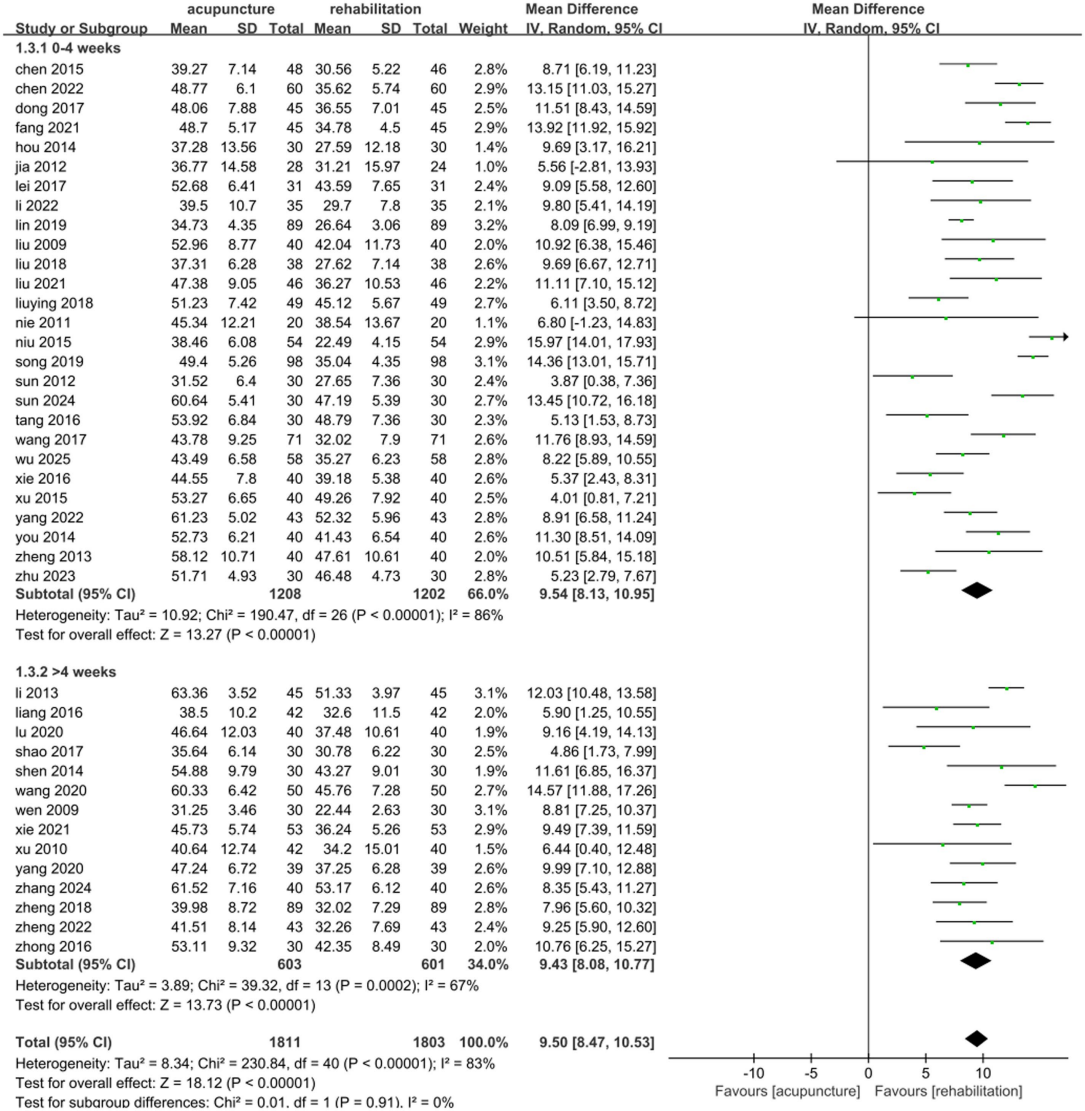
Forest plot of the treatment duration subgroup of acupuncture treatment combined with Rehab vs. on FMA.

To evaluate the effects of acupuncture type and treatment duration on combined data, subgroup analyses were conducted. Acupuncture type subgroups included MA, EA, and WA. No significant differences were observed between subgroups (*p* = 0.99, *I*^2^ = 0%). WA combined with Rehab showed reduced heterogeneity and significant motor function improvement (MD: 9.48, 95% CI: [8.28, 10.67], *p* < 0.00001; Figure 4). Eleven studies (31, 33–37, 40, 49, 58, 64, 66) comparing EA plus Rehab with Rehab alone demonstrated significant motor function enhancement (MD: 9.64, 95% CI: [7.64, 11.64], *p* < 0.00001; Figure 4). Similarly, 22 studies (25–27, 38, 39, 41, 43, 45, 46, 48, 50–54, 56, 57, 60, 61, 65, 67, 69, 70) comparing MA plus Rehab with Rehab alone also showed significant improvement (MD: 9.44, 95% CI: [8.01, 10.87], *p* < 0.00001; Figure 4). For treatment duration subgroups (0–4 weeks vs. >4 weeks), no significant differences were found (*p* = 0.91, *I*^2^ = 0%). Both intervals improved motor function compared to controls (0–4 weeks: MD 9.54, 95% CI: [8.13, 10.95], *p* < 0.00001; >4 weeks: MD 9.43, 95% CI: [8.07, 10.77], *p* < 0.000001; Figure 5). Sensitivity analysis by removing high-weight studies identified Wang (54) in the >4 weeks subgroup as a potential heterogeneity source, likely due to its extended treatment duration. Exclusion of this study stabilized results with reduced heterogeneity (Supplementary Table 1). In acupuncture-type subgroup sensitivity analyses, no individual study significantly influenced MD values or heterogeneity.

#### 3.5.2 VAS

**Figure 6:**
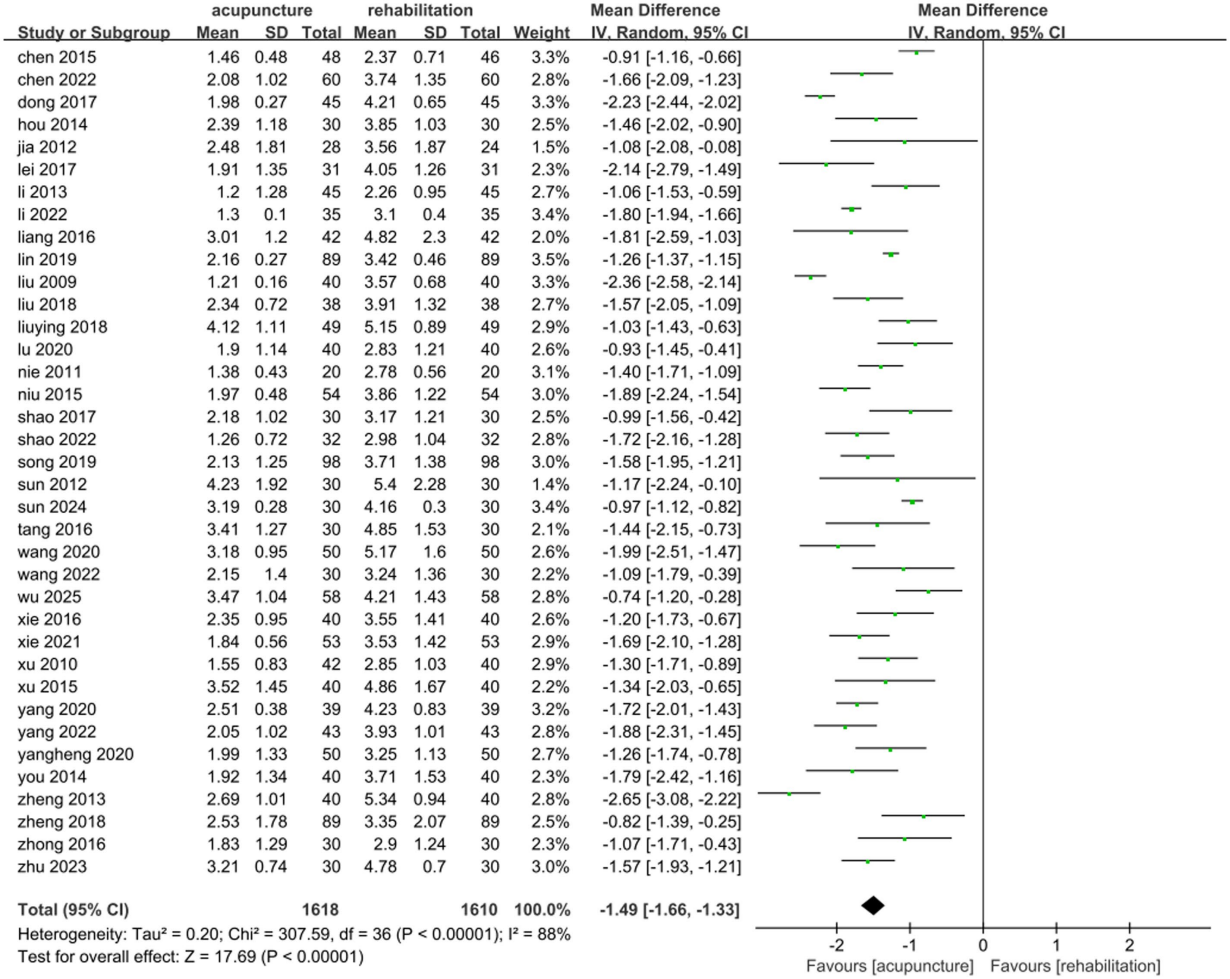
Forest plot of acupuncture treatment combined with Rehab vs. Rehab on VAS.

**Figure 7:**
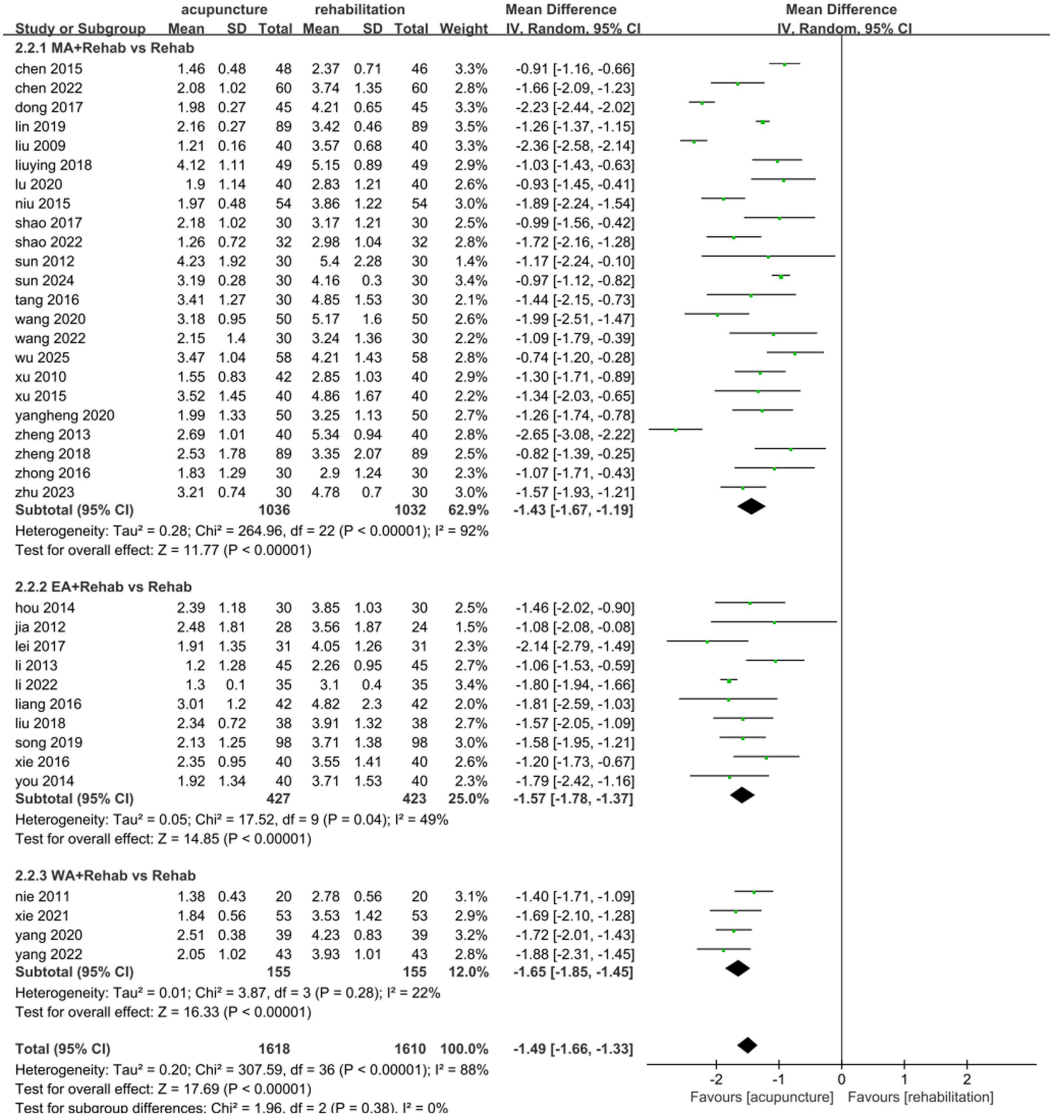
Forest plot of the intervention subgroup of acupuncture treatment combined with Rehab vs. Rehab on VAS.

**Figure 8:**
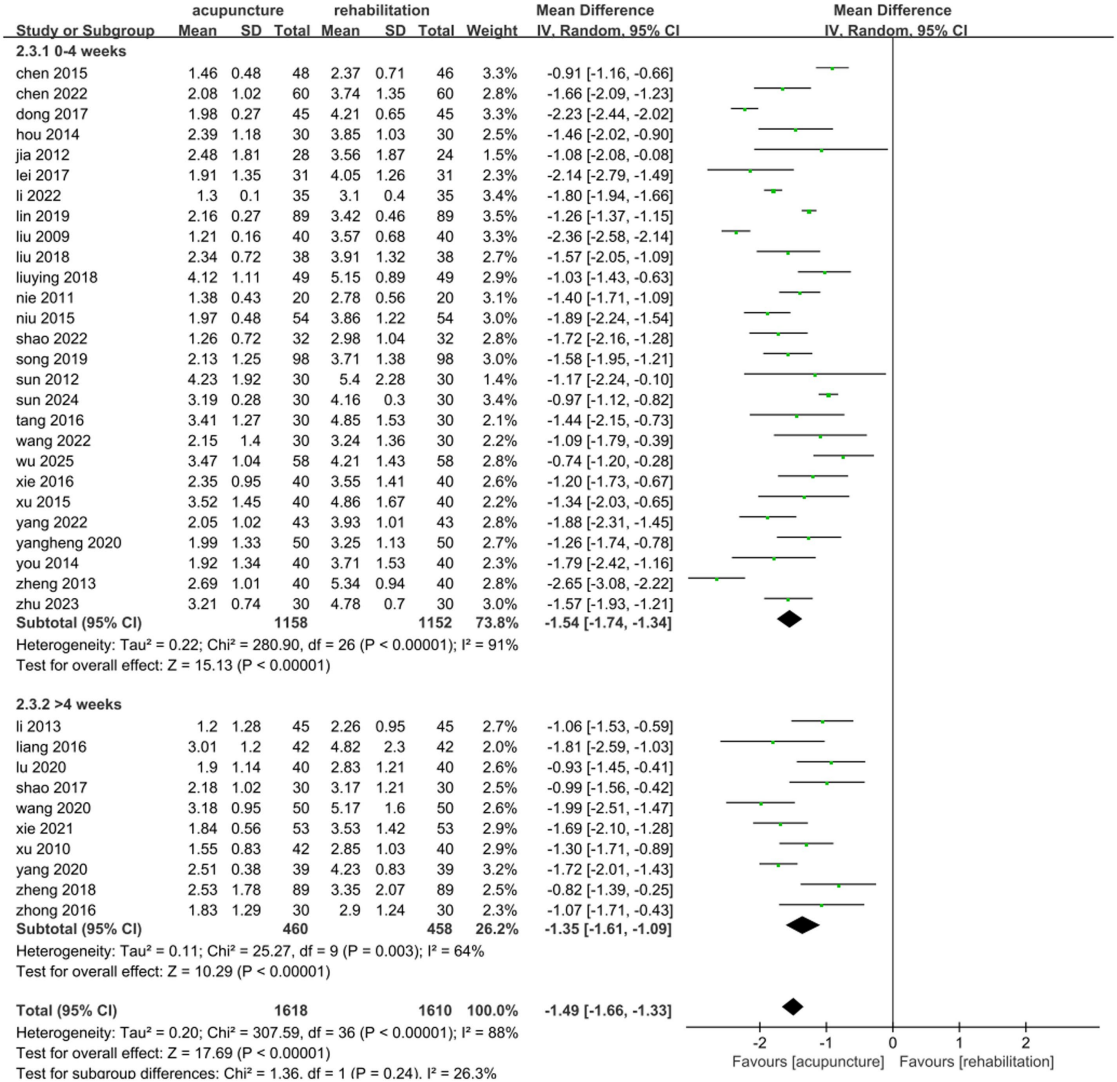
Forest plot of the treatment duration subgroup of acupuncture treatment combined with Rehab vs. on VAS.

Thirty-seven studies (25, 26, 31, 33–41, 43–47, 49–52, 54, 55, 57–65, 67, 69–71) involving 3,228 patients demonstrated that acupuncture combined with Rehab significantly reduced VAS scores compared to Rehab alone, with a pooled MD of −1.49 (95% CI: [−1.66, −1.33]; *Z* = 17.69, *p* < 0.00001) using a random-effects model. Substantial heterogeneity was observed (*p* < 0.00001, *I*^2^ = 86%)(Figure 6). Subgroup analyses by acupuncture type and treatment duration showed no significant inter-subgroup differences (acupuncture method: *p* = 0.38, *I*^2^ = 0%; duration: *p* = 0.24, *I*^2^ = 26.3%). Both EA + Rehab (MD: −1.57, 95% CI: [−1.78, −1.37], *p* < 0.0001) and WA + Rehab (MD: −1.65, 95% CI: [−1.85, −1.45]) exhibited reduced heterogeneity, while MA + Rehab also showed significant improvement (MD: −1.43, 95% CI: [−1.67, −1.19], *p* < 0.00001) (Figure 7). Treatment durations of 0–4 weeks (MD: −1.54, 95% CI: [−1.74, −1.34]) and > 4 weeks (MD: −1.35, 95% CI: [−1.67, −1.19]) both achieved statistical significance (*p* < 0.00001) (Figure 8). Sensitivity analysis excluding Li 2013 (35) mitigated heterogeneity in the EA + Rehab subgroup, possibly due to variations in disease duration and acupoint selection, though results remained favorable for combination therapy (Supplementary Table 1).

#### 3.5.3 ADL

**Figure 9:**
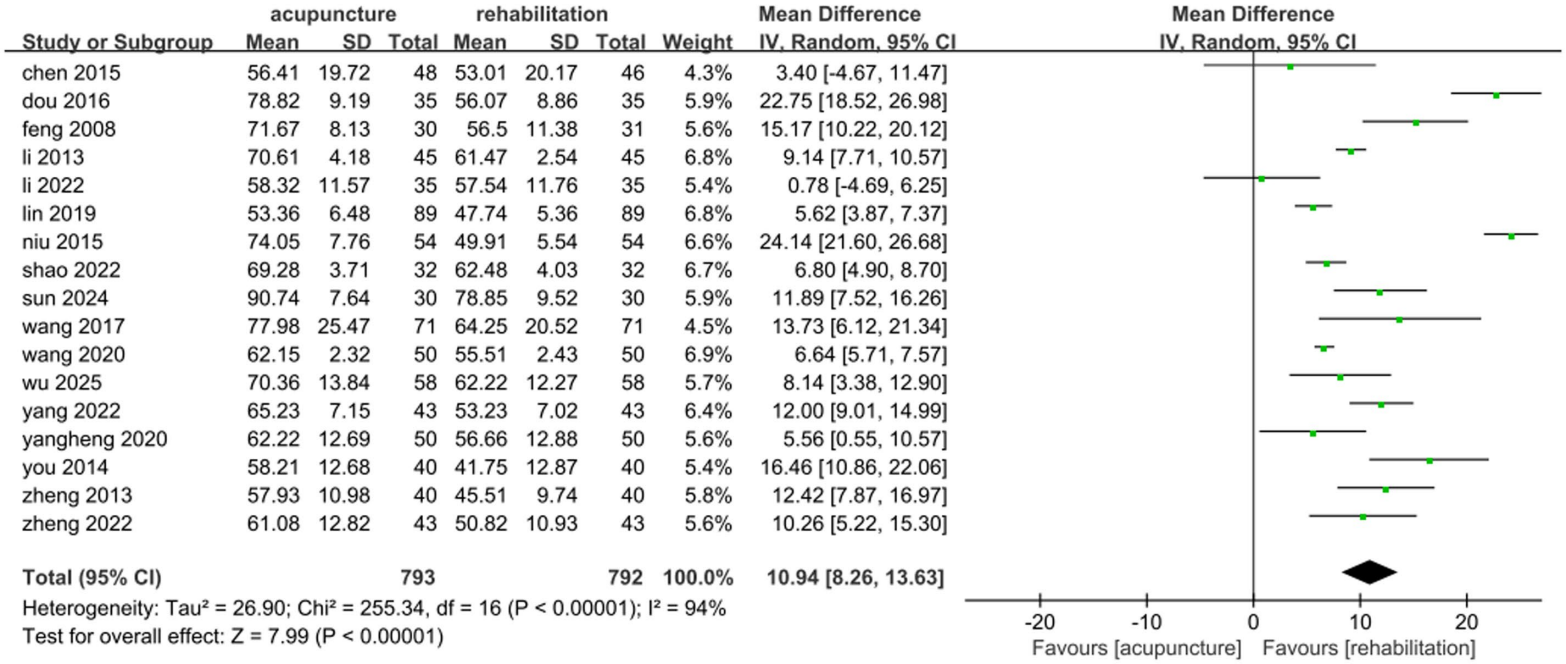
Forest plot of acupuncture treatment combined with Rehab vs. Rehab on ADL.

**Figure 10:**
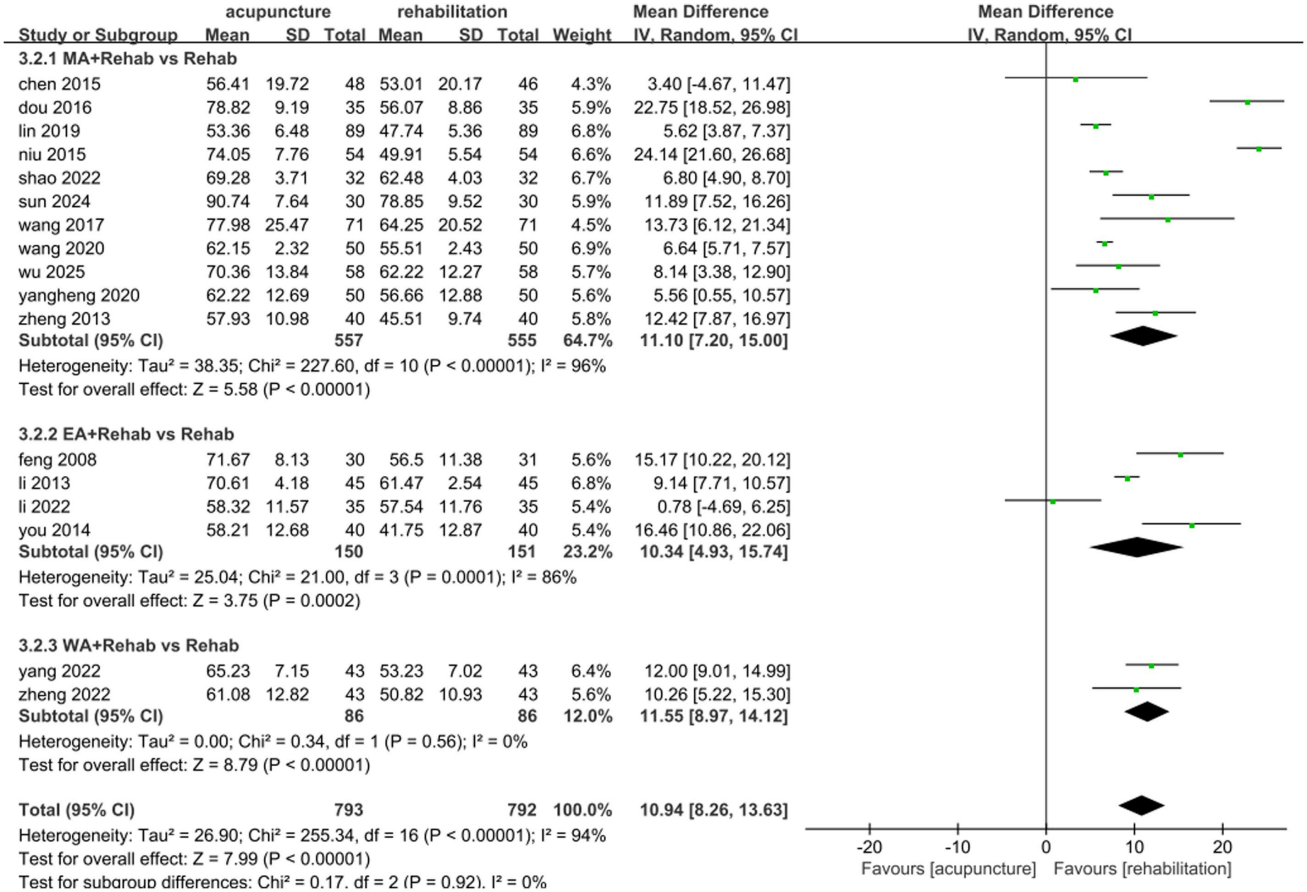
Forest plot of the intervention subgroup of acupuncture treatment combined with Rehab vs. Rehab on ADL. MA, manual acupuncture; Ea, electro-acupuncture; MA, warm acupuncture; Rehab, rehabilitation.

**Figure 11:**
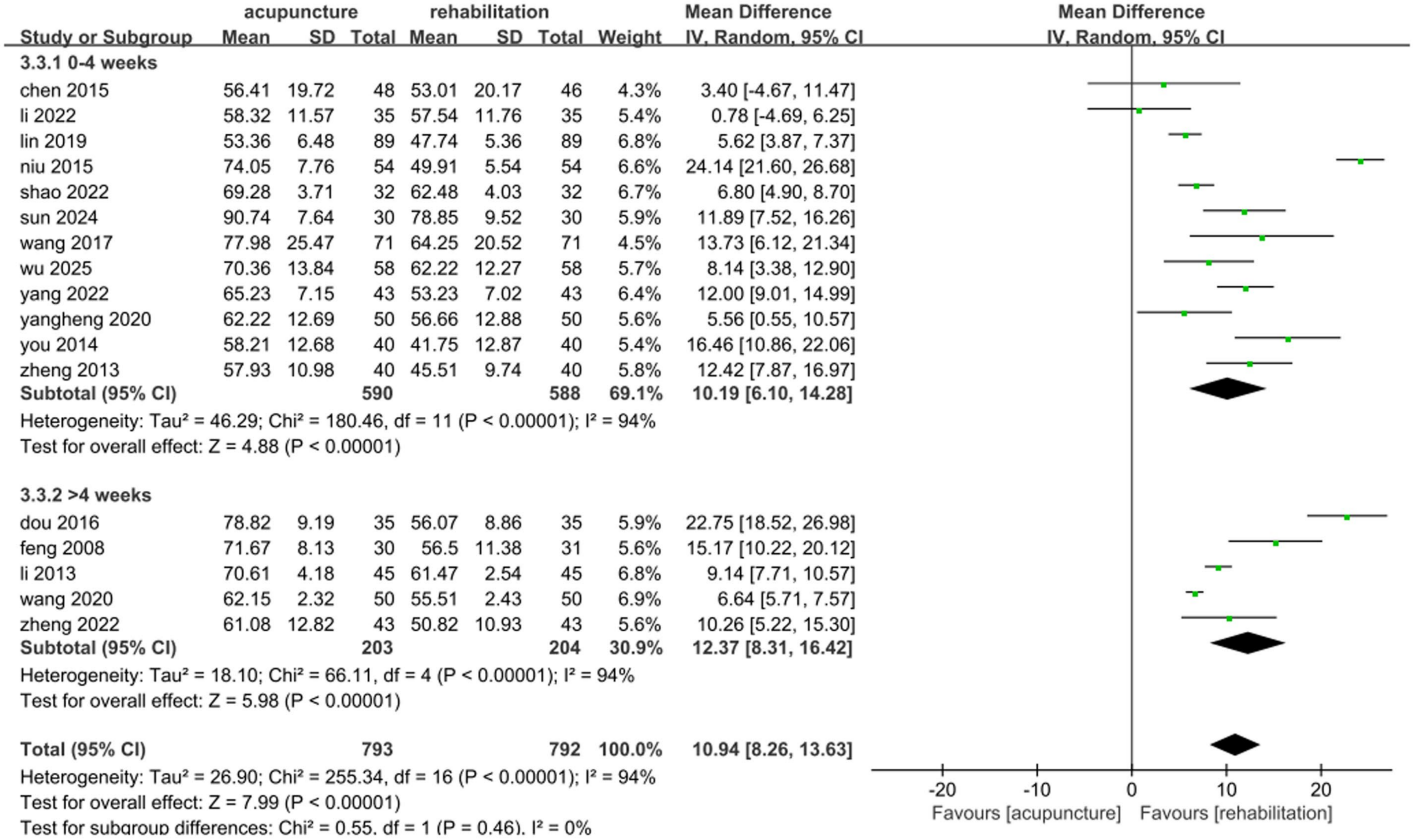
Forest plot of the treatment duration subgroup of acupuncture treatment combined with Rehab vs. on ADL.

A total of 1,585 patients across 17 studies (25, 28, 30, 35, 36, 38, 45, 47, 51, 53, 54, 57, 63–65, 68, 71) evaluated the effects of acupuncture combined with Rehab on ADL using the Barthel Index (BI) or modified Barthel Index (MBI). A random-effects model was applied due to significant heterogeneity (*p* < 0.00001, *I*^2^ = 94%). The meta-analysis demonstrated that acupuncture combined with Rehab significantly improved self-care ability compared to controls (MD: 10.94, 95% CI: 8.26–13.63, *p* < 0.00001, see Figure 9). Subgroup analyses by acupuncture modality revealed superior outcomes for manual acupuncture + Rehab (MD: 11.10, 95% CI: 7.20–15.00, *p* < 0.00001), electroacupuncture + Rehab (MD: 10.34, 95% CI: 4.93–15.74, *p* = 0.0002), and warm acupuncture + Rehab (MD: 11.55, 95% CI: 8.97–14.12, *p* < 0.00001, see Figure 10) over Rehab alone. Treatment duration subgroup analyses showed consistent benefits for acupuncture + Rehab across both short-term (0–4 weeks: MD: 10.19, 95% CI: 6.10–14.28, *p* < 0.0001) and extended periods (>4 weeks: MD: 12.37, 95% CI: 8.31–16.42, *p* < 0.0001, see Figure 11). The results of the sensitivity analysis showed that the exclusion of either study had little effect on the MD values of the pooled data, and we were unable to find a clear reason for the heterogeneity. Differences in acupoint selection, acupuncture retention time, and treatment frequency in the included studies may be potential reasons for the heterogeneity.

#### 3.5.4 Edema

Sufficient information on edema was detected in 476 patients with SHS across 4 studies (26, 43, 49, 61). A random-effects model analysis revealed a statistically significant difference between acupuncture therapy combined with Rehab and Rehab alone (MD: −0.65, 95% CI: [−0.93, −0.38], *p* < 0.00001; see Supplementary Figure 1). Subgroup analysis based on acupuncture methods demonstrated that MA combined with Rehab significantly reduced edema (MD: −0.55, 95% CI: [−0.94, −0.16], *p* < 0.00001; see Supplementary Figure 2). Further subgroup analysis stratified by treatment duration (0–4 weeks) showed similar results (MD: −0.76, 95% CI: [−1.52, −1.01], *p* < 0.00001; see Supplementary Figure 3). Heterogeneity in the MA + Rehab subgroup was notably reduced after excluding Chen (26), potentially attributed to variations in disease duration and acupoint selection. Xu (61) was a key source of heterogeneity in the 0–4 week subgroup, likely attributed to older participants and longer disease duration. After excluding this study, pooled results remained significantly in favor of acupuncture combined with Rehab (Supplementary Table 1).

## 4 Adverse events

By scanning 47 studies, we found that the majority of patients with adverse events were able to recover spontaneously without medical intervention. The verse events are described as follows: Three studies (26, 45, 60) reported no adverse events during the treatment period. Two studies (36, 54) reported two patients with subcutaneous hemorrhage, compression relief, and pain 5, redness 4, and pruritus 3. No adverse events were mentioned in the remaining studies. The data combined by fixed effects model showed that there was no significant difference in the occurrence of adverse reactions and Rehab (RR: 1.15, 95% CI:[0.42, 3.14], *I*^2^ = 0%, *p* = 0.78, see Supplementary Figure 4).

## 5 Publication bias

Funnel plot and Egger’s test were used to evaluate publication bias based on the FMA, VAS and ADL scales. The distribution of the funnel plot is asymmetric, with some studies exceeding the 95% confidence interval (see Supplementary Figures 5–7). Egger’s test showed no publication bias in the FMA (*p* = 0.126), VAS (*p* = 0.896), and ADL scales (*p* = 0.133) (see Supplementary Tables 2–4). The test results of the funnel plot show potential publication bias.

## 6 Evidence assessment of outcome measures

The strength of evidence for the above four scales was assessed as a ‘low’ level of certainty. Defects in the study design and considerable statistical heterogeneity were the main reasons for reduced evidence certainty (see Table 3).

**Table 3:** Results of evidence assessment.

Quality assessment							No of patients		Effect		Quality	Importance
No of studies	Design	Risk of bias	Inconsistency	Indirectness	Imprecision	Other considerations	Acupuncture therapy	Rehabilitation	Relative (95% CI)	Absolute		
FMA												
41	Randomized trials	Serious^a^	Serious^b^	No serious	Not serious	None	1811	1803	-	MD 9.50 higher (8.47–10.53 higher)	⊕ ⊕ OO Low	Critical
VAS												
37	Randomized trials	Seriousa^a^	Serious^b^	No serious	Not serious	None	1,618	1,610	-	MD -1.49 higher (−1.66 to −1.33 higher)	⊕ ⊕ OO Low	Critical
ADL												
17	Randomized trials	Serious^a^	Serious^b^	No serious	Not serious	None	793	792	-	MD 10.94 higher (8.26–13,63 higher)	⊕ ⊕ OO Low	Important
Edema												
4	Randomized trials	Serious^a^	Serious^b^	No serious	Not serious	None	238	238	-	MD -0.65 higher (−0.93 to −0.38 higher)	⊕ ⊕ OO Low	Important

a Inadequate description of allocation concealment and failure to implement or describe blinding of participants and personnel.

b Considerable statistical heterogeneity.

## 7 Discussion

In this systematic review, 47 randomized controlled trials involving 4,129 patients were included. Our findings from this review suggest that acupuncture therapy combined with Rehab benefits motor function (FMA), pain (VAS), and activities of daily living (ADL) in SHS patients. The subgroup analysis results showed no difference in the upper limb motor function improvement by manual acupuncture, electric acupuncture, and warm acupuncture (measured by FMA, VAS, and ADL). In terms of treatment duration, the results did not change with increasing treatment duration (as measured by FMA, VAS, and ADL). Two studies (36, 54) (4%) reported AEs related to acupuncture treatment, with the main AEs including subcutaneous hemorrhage, pain, itching, and redness. Sensitivity analysis showed that the effect of acupuncture combined with Rehab was robust to improving motor function, reducing pain and improving daily living ability in SHS patients. The included studies had methodological flaws and high heterogeneity, giving a “low” level of certainty of evidence.

The mechanism of acupuncture to relieve pain have been extensively studied. Modern neurological studies believe that the analgesic effect of acupuncture is mainly based on the effect of acupuncture on the nervous system and neurotransmitters. Acupuncture at acupuncture points plays an important role in the release of pain-reducing signaling molecules such as opioid peptides, glutamate and calcium adenosine (72). Acupuncture treatment can effectively regulate the levels of pro-inflammatory and anti-inflammatory factors TNF-A, IL-13 and IL-6, and can also reduce the inflammatory immune response and effectively relieve pain by inhibiting the NLRP 3 inflammasome and its inflammatory factors Caspase-1 and IL-13 (73, 74). Numerous studies have shown that phosphorylation of ipsilateral extracellular signal-regulated kinases (ERKs) and subsequent CREB activation, and various calmodulin kinases such as CamKV and cAMP/protein kinase A (PKA) transfer to neurons and regulate the activity of transcriptional regulators. It is suggested that acupuncture may lead to changes in neuronal and synaptic morphological structures that may affect the plasticity of hippocampal synapses (75). In motor function, acupuncture mainly by adjusting the bilateral motor cortex, auxiliary motor area, central back, the pole, lingual gyrus, cerebellum, frontal gyrus, and the precuneus area function connection, adjust the left prefrontal network, default mode network and sensorimotor network connection conduction mode, change the network between information input and output way, thus improving patients motor function (76–78). Currently, there is no consensus on the mechanism of action of acupuncture for SHS or CRPS, so how acupuncture affects sympathetic/somatic nervous system dysfunction needs further investigation.

However, before applying the study results to clinical practice, we must consider some of the limitations in this review. First, although 47 RCTs items were ultimately included in the mate analysis, the random sequence generation method for most studies was ambiguous, the lack of blindness among participants and operators, and blind uncertainty among outcome assessors. Specifically, 40.4% of the studies did not explicitly address the method for random sequence generation. None of the RCTs implemented allocation concealment. Participants and acupuncturists were not blinded in any of the studies. Blinding of outcome assessors in all studies was assessed as an “unclear” risk of bias because of a lack of information. These factors will inevitably lead to a degree of selection bias, detection bias, and reporting bias. Second, another limitation is the high degree of heterogeneity between the included studies. Although we sought to identify the cause of high heterogeneity by subgroup analysis and sensitivity analysis, acupuncture, as a complex treatment, may itself be a source of heterogeneity. In order to maximize the benefits from acupuncture treatment, we need to consider a combination of factors such as the choice and combination of acupuncture points, the depth and retention time of needles, and the duration and frequency of treatment. Although we grouped the studies by acupuncture modality and treatment duration, these parameters were still variable, and we were unable to accurately and reliably determine the reasons for the significant heterogeneity. Therefore, future experimental studies should elaborate on the acupuncture protocol to further improve the reporting completeness. Furthermore, variability in the skill level of acupuncture therapists can also have an impact on treatment outcomes. The variability of these factors may be responsible for the high heterogeneity. These two limitations are the main reasons for the lower level of evidence in this review. Furthermore, most of the studies in this review were published and only one study was published in the English database. Therefore, the superior efficacy of acupuncture for SHS over rehabilitation needs to be interpreted cautiously in China. Patients with SHS have a long-term recovery period, but all studies used short-term prognostic indicators and lack long-term follow-up of patients. Therefore, the long-term efficacy of acupuncture for SHS needs to be further explored. Furthermore, we should note that in this review, outcome measures such as FMA, VAS and ADL were used as screening tools for recovery in SHS patients rather than as a comprehensive assessment of symptom relief. A further limitation is that the severity of SHS patient staging was not clearly defined according to the inclusion criteria. Therefore, it is suggested that future studies can be conducted in specific areas to provide more definite and high-quality evidence for the efficacy of acupuncture for SHS. Finally, among the 47 included studies, three studies reported no adverse events during acupuncture treatment, and two studies reported mild adverse events in 16 patients. Acupuncture treatment appears to be relatively safe and does not cause serious AEs.

Due to the unique geographical characteristics of acupuncture treatment, we had to look for RCTs related to acupuncture treatment as comprehensively as possible in Chinese journals. All of the studies included in this review were conducted at the Chinese mainland. Further studies should be conducted around the world to involve more ethnic and culturally diverse populations. However, we note that the vast majority of studies published in Chinese databases suffer from experimental design flaws. In order to truly effectively evaluate the efficacy of acupuncture for SHS and to provide high quality evidence for clinical practice, future studies need to be more rigorous in experimental design and methodology. As a non-pharmacological intervention, acupuncture therapists need to choose different acupoints for treatment in clinical practice, which makes it difficult to achieve operator blinding. However, we could achieve blinding to participants by sham or placebo acupuncture to reduce the occurrence of bias. Furthermore, blinding of the outcome assessors is also feasible and necessary.

## 8 Conclusion

This systematic review suggests that the addition of acupuncture treatment in rehabilitation training may have a positive effect on improving motor function, reducing pain and improving daily life in SHS patients with SHS. However, due to the methodological limitations of the included studies, this evidence was graded as “low ‘, and the results should be treated with caution. Future clinical studies should use a high-quality randomized, double-blind controlled trial design. Meanwhile, long-term follow-up and efficacy assessment and clinical studies with large-scale multicenter sample sizes are highly desirable.

## Data Availability

The original contributions presented in the study are included in the article/Supplementary material, further inquiries can be directed to the corresponding author.

## Supplementary material

SUPPLEMENTARY FIGURE 1 Forest plot of acupuncture treatment combined with Rehab vs. Rehab on Edema.

SUPPLEMENTARY FIGURE 2 Forest plot of the intervention subgroup of acupuncture treatment combined with Rehab vs. Rehab on Edema MA, manual acupuncture; EA, electro-acupuncture; Rehab, rehabilitation.

SUPPLEMENTARY FIGURE 3 Forest plot of the treatment duration subgroup of acupuncture treatment combined with Rehab vs. on Edema.

SUPPLEMENTARY FIGURE 4 The forest plot of acupuncture treatment combined with Rehab vs. Rehab on the incidence of adverse events.

SUPPLEMENTARY FIGURE 5 The funnel plot of FMA.

SUPPLEMENTARY FIGURE 6 The funnel plot of VAS.

SUPPLEMENTARY FIGURE 7 The funnel plot of ADL.
