# Supplementary material for: Acupuncture versus rehabilitation for post-stroke shoulder-hand syndrome: a systematic review and meta-analysis of randomized controlled trials
Source: Front Neurol. 2025 Apr 2;16:1488767. doi: 10.3389/fneur.2025.1488767 (PMC12000064; doi:10.3389/fneur.2025.1488767)
Supplement: SUPPLEMENTARY FIGURE 1 — Forest plot of acupuncture treatment combined with Rehab vs. Rehab on Edema. [file Data_Sheet_1.zip › Supplementary Appendix.docx]

**Appendix:** search strategies for databases

**PubMed search strategy**

| Strategies |  |
| --- | --- |
| #1 | "Acupuncture"[Mesh] OR "Acupuncture Therapy"[Mesh] OR "Acupuncture Points"[Mesh] |
| #2 | “Acupuncture” [Title/Abstract]) OR (Acupuncture Therapy [Title/Abstract])) OR (Acupuncture Points [Title/Abstract])) OR (Electroacupuncture [Title/Abstract])) OR (Warm acupuncture [Title/Abstract])) OR (Scalp acupuncture [Title/Abstract] |
| #3 | (#1 OR #2) |
| #4 | **Reflex Sympathetic Dystrophy** [Mesh] |
| #5 | “**Shoulder-hand syndrome**” [Title/Abstract]) OR (CRPS Type I [Title/Abstract])) |
| #6 | (#4 OR #5) |
| #7 | "Stroke"[Mesh] OR "Stroke, Lacunar"[Mesh] OR "Hemorrhagic Stroke"[Mesh] OR "Embolic Stroke"[Mesh] OR "Thrombotic Stroke"[Mesh] OR "Ischemic Stroke"[Mesh] |
| #8 | stroke[Title/Abstract]) OR (stroke, lacunar [Title/Abstract])) OR (hemorrhagic stroke[Title/Abstract])) OR (embolic stroke[Title/Abstract])) OR (thrombotic stroke[Title/Abstract])) OR (ischemic stroke[Title/Abstract])) OR (cerebrovascular accident[Title/Abstract])) OR (cerebrovascular apoplexy[Title/Abstract])) OR (apoplexy[Title/Abstract])) OR (lacunar stroke[Title/Abstract])) OR (lacunar syndrome[Title/Abstract])) OR (lacunar infarction[Title/Abstract])) OR (intracerebral hemorrhagic stroke[Title/Abstract])) OR (intracerebral hemorrhage stroke[Title/Abstract])) OR (acute ischemic stroke[Title/Abstract] |
| #9 | (#7 OR #8) |
| #10 | (#3 AND #6 AND #9) |

**Web of science search strategy**

1.**(ALL=(Reflex Sympathetic Dystrophy)) OR ALL=(shoulder hand syndrome)**

1. **(((((((((((((ALL=(Stroke)) OR ALL=(Stroke, Lacunar)) OR ALL=(Hemorrhagic Stroke)) OR ALL=(Embolic Stroke)) OR ALL=(Thrombotic Stroke)) OR ALL=(Ischemic Stroke)) OR ALL=(cerebrovascular accident)) OR ALL=(cerebrovascular apoplexy)) OR ALL=(apoplexy)) OR ALL=(lacunar stroke)) OR ALL=(lacunar syndrome)) OR ALL=(lacunar infarction)) OR ALL=(intracerebral hemorrhagic stroke)) OR ALL=(acute ischemic stroke)**
2. **(((((ALL=(Acupuncture)) OR ALL=(Acupuncture Therapy)) OR ALL=(Acupuncture Points)) OR ALL=(Acupuncture, Ear)) OR ALL=(Electroacupuncture)) OR ALL=(Scalp acupuncture))OR ALL=(Warm acupuncture)**
3. **1 AND 2 AND 3**

**Embase search strategy**

(acupuncture:ab,ti OR 'acupuncture therapy':ab,ti OR electroacupuncture:ab,ti OR 'warm acupuncture':ab,ti OR 'acupuncture points':ab,ti OR 'scalp acupuncture':ab,ti) AND ('**Reflex Sympathetic Dystrophy**':ab,ti OR '**shoulder hand syndrome**':ab,ti OR 'SHS':ab,ti OR 'CRPS Type I':ab,ti) AND (stroke:ab,ti OR stroke,lacunar:ab,ti OR 'hemorrhagic stroke':ab,ti OR 'embolic stroke':ab,ti OR 'thrombotic stroke':ab,ti OR 'ischemic stroke':ab,ti OR 'cerebrovascular accident':ab,ti OR 'cerebrovascular apoplexy':ab,ti OR apoplexy:ab,ti OR 'lacunar stroke':ab,ti OR 'lacunar syndrome':ab,ti OR 'lacunar infarction':ab,ti OR 'intracerebral hemorrhagic stroke':ab,ti OR 'intracerebral hemorrhage stroke':ab,ti OR 'acute ischemic stroke':ab,ti)

**Cochrane library search strategy**

(acupuncture OR acupuncture therapy OR electroacupuncture OR warm acupuncture OR acupuncture points OR scalp acupuncture):ti,ab,kw AND (**Reflex Sympathetic Dystrophy** OR **shoulder hand syndrome** OR SHS OR CRPS Type I):ti,ab,kw AND (stroke OR stroke, lacunar OR hemorrhagic stroke OR embolic stroke OR thrombotic stroke OR ischemic stroke OR cerebrovascular accident OR cerebrovascular apoplexy OR apoplexy OR lacunar stroke OR lacunar syndrome OR lacunar infarction OR intracerebral hemorrhagic stroke OR intracerebral hemorrhage stroke OR acute ischemic stroke):ti,ab,kw

Chinese Biological Medicine Database search strategy

1 "针灸疗法"[不加权:扩展] OR "针刺疗法"[不加权:扩展] OR "毫针"[不加权:扩展]

2"针刺疗法"[全部字段:智能] OR "针灸疗法"[全部字段:智能] OR "毫针"[全部字段:智能] OR "电针"[全部字段:智能] OR "头针"[全部字段:智能] OR "温针灸"[全部字段:智能] OR "体针"[全部字段:智能] OR "针灸"[全部字段:智能] OR "针刺"[全部字段:智能]

3 (#1) OR (#2)

4 "卒中"[不加权:扩展]) OR "中风"[不加权:扩展]) OR "脑血管障碍"[不加权:扩展]

5 "中风"[全部字段:智能] OR "卒中"[全部字段:智能] OR "脑栓塞"[全部字段:智能] OR "脑卒中"[全部字段:智能] OR "脑梗塞"[全部字段:智能] OR "脑梗死"[全部字段:智能] OR "脑出血"[全部字段:智能] OR "脑血管意外"[全部字段:智能] OR "脑血管病"[全部字段:智能]

6 (#4) OR (#5)

7"肩手综合征"[不加权:扩展]

8"反射性交感神经营养障碍"[全部字段:智能]

9 (#4) OR (#8)

10 (#3) AND (#6) AND (#9)

China National Knowledge Infrastructure (CNKI) database search strategy

（主题：针灸疗法 + 针灸疗法 +毫针（精确）） OR （篇摘要：针刺疗法 + 针灸疗法 + 温针灸 + 体针 + 头针 + 电针 + 针灸 + 针刺（精确）） AND （主题：中风 + 卒中 + 脑血管障碍（精确）） OR （篇摘要：中风 + 卒中 +脑血管障碍 + 脑梗塞 + 脑梗死 + 脑出血 + 脑卒中 + 脑栓塞 + 脑血管病 + 脑血管意外（精确）） AND （主题：肩手综合征（精确）） OR （篇关摘：反射性交感神经营养障碍）

Chinese Science and Technology periodical Database (VIP) search strategy

（题名或关键词=针刺疗法 OR 针灸疗法 OR 毫针 OR 电针 OR 体针 OR 头针 OR 温针灸 OR 针刺 OR 针灸) AND （题名或关键词=卒中 OR 中风 OR 脑血管障碍 OR 脑卒中 OR 脑梗死 OR 脑梗塞 OR 脑出血 OR 脑栓塞 OR 脑血管病 OR 脑血管病意外) AND （题名或关键词=肩手综合征 OR 反射性交感神经营养不良）

Wan fang Database search strategy

题名或关键词：（针刺疗法 OR 针灸疗法 OR 毫针 OR 电针 OR 体针 OR 头针 OR 温针灸 OR 针刺 OR 针灸） AND 题名或关键词：（卒中 OR 中风 OR 脑血管障碍 OR 脑卒中 OR 脑梗死 OR 脑梗塞 OR 脑出血 OR 脑栓塞 OR 脑血管病 OR 脑血管病意外） AND 题名或关键词：（肩手综合征 OR 反射性交感神经营养不良）
