# Supplementary material for: Acupuncture versus rehabilitation for post-stroke shoulder-hand syndrome: a systematic review and meta-analysis of randomized controlled trials
Source: Front Neurol. 2025 Apr 2;16:1488767. doi: 10.3389/fneur.2025.1488767 (PMC12000064; doi:10.3389/fneur.2025.1488767)
Supplement: SUPPLEMENTARY FIGURE 1 — Forest plot of acupuncture treatment combined with Rehab vs. Rehab on Edema. [file Data_Sheet_1.zip › Supplementary Table.docx]

**Supplementary Table 1** Results of the sensitivity analyses

| Study | Subgroup | MD(95%CI) | Tau^2^ | I^2^ | P |
| --- | --- | --- | --- | --- | --- |
| FMA |  |  |  |  |  |
| Wang 2020 | >4 weeks | 9.03[7.98,10.22] | 2.27 | 54 | 0.01 |
| VAS |  |  |  |  |  |
| Li 2013 | EA+Rehab | -1.67[-1.84,-1.50] | 0.01 | 20 | 0.26 |
| Edema |  |  |  |  |  |
| Chen 2022 | MA+Rehab | -0.37[-0.55,0.20] | 0 | 0 | 0.37 |
| Xu 2015 | 0-4 weeks | -0.90[0.99,-0.81] | 0 | 0 | 0.84 |

**Supplementary Table 2.** Tests for Publication Bias of FMA (Egger’s test)

| Std_Eff | Coef. | Std.Err. | t | P>\|t\| | [95% Conf. Interval] | |
| --- | --- | --- | --- | --- | --- | --- |
| slope | 11.71783 | 1.164695 | 10.06 | 0.000 | 9.362015 | 14.07365 |
| bias | -1.396857 | .8923525 | -1.57 | 0.126 | -3.20181 | .4080961 |

**Supplementary Table 3.** Tests for Publication Bias of VAS (Egger’s test)

| Std_Eff | Coef. | Std.Err. | t | P>\|t\| | [95% Conf. Interval] | |
| --- | --- | --- | --- | --- | --- | --- |
| slope | -1.482038 | .1425036 | -10.40 | 0.000 | -1.771336 | -1.192741 |
| bias | -.1181521 | .8998419 | -0.13 | 0.896 | -1.944928 | 1.708624 |

**Supplementary Table 4.** Tests for Publication Bias of ADL (Egger’s test)

| Std_Eff | Coef. | Std.Err. | t | P>\|t\| | [95% Conf. Interval] | |
| --- | --- | --- | --- | --- | --- | --- |
| slope | 6.335275 | 1.934653 | 3.27 | 0.005 | 2.21166 | 10.45889 |
| bias | 2.50152 | 1.575007 | 1.59 | 0.133 | -.855529 | 5.858569 |
