# Supplementary figures and images for: Acupuncture versus rehabilitation for post-stroke shoulder-hand syndrome: a systematic review and meta-analysis of randomized controlled trials
Source: Front Neurol. 2025 Apr 2;16:1488767. doi: 10.3389/fneur.2025.1488767 (PMC12000064; doi:10.3389/fneur.2025.1488767)

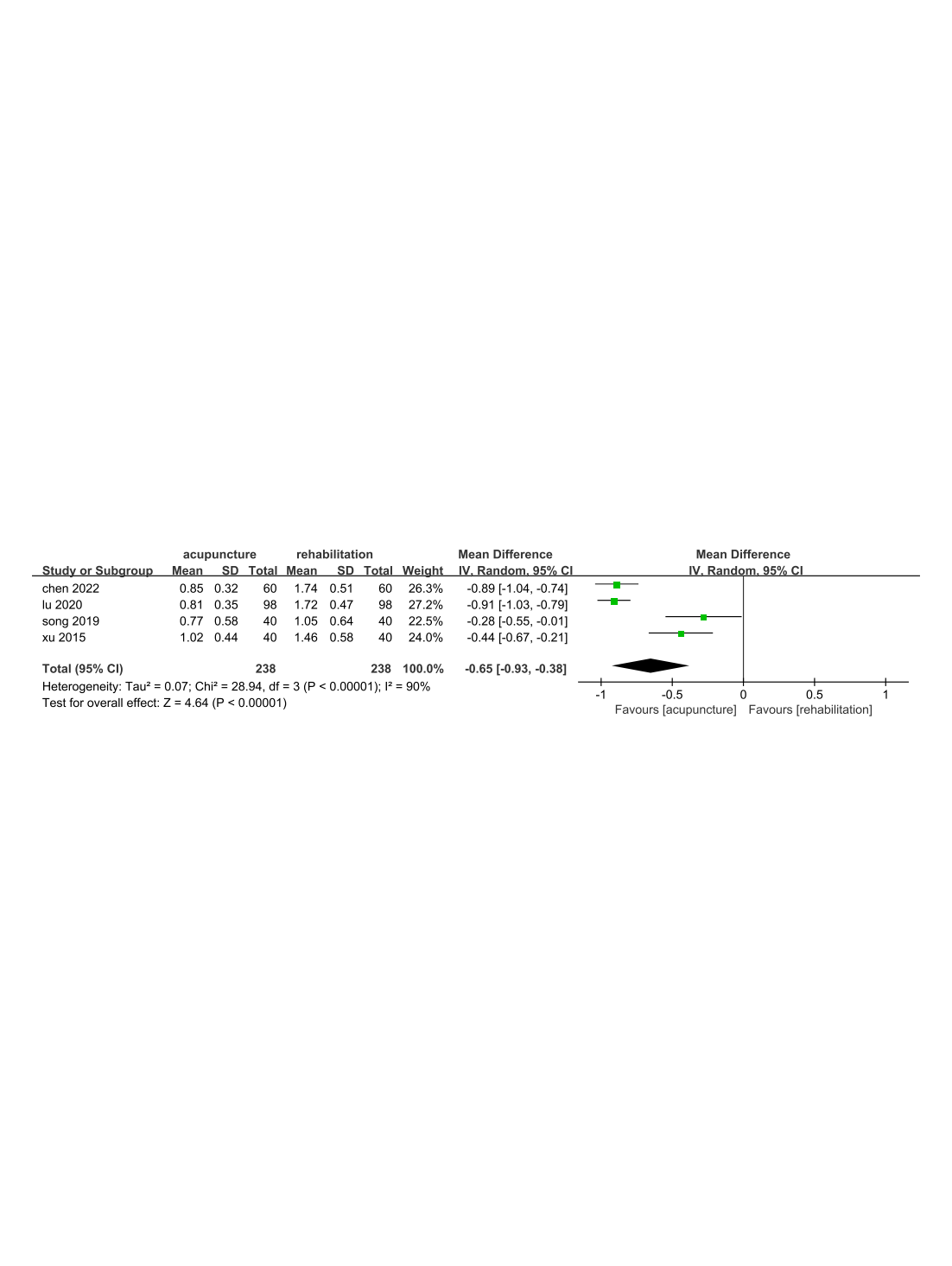

Supplement: SUPPLEMENTARY FIGURE 1 — Forest plot of acupuncture treatment combined with Rehab vs. Rehab on Edema. [file Data_Sheet_1.zip › Figure/Supplementary Figure 1.tif]

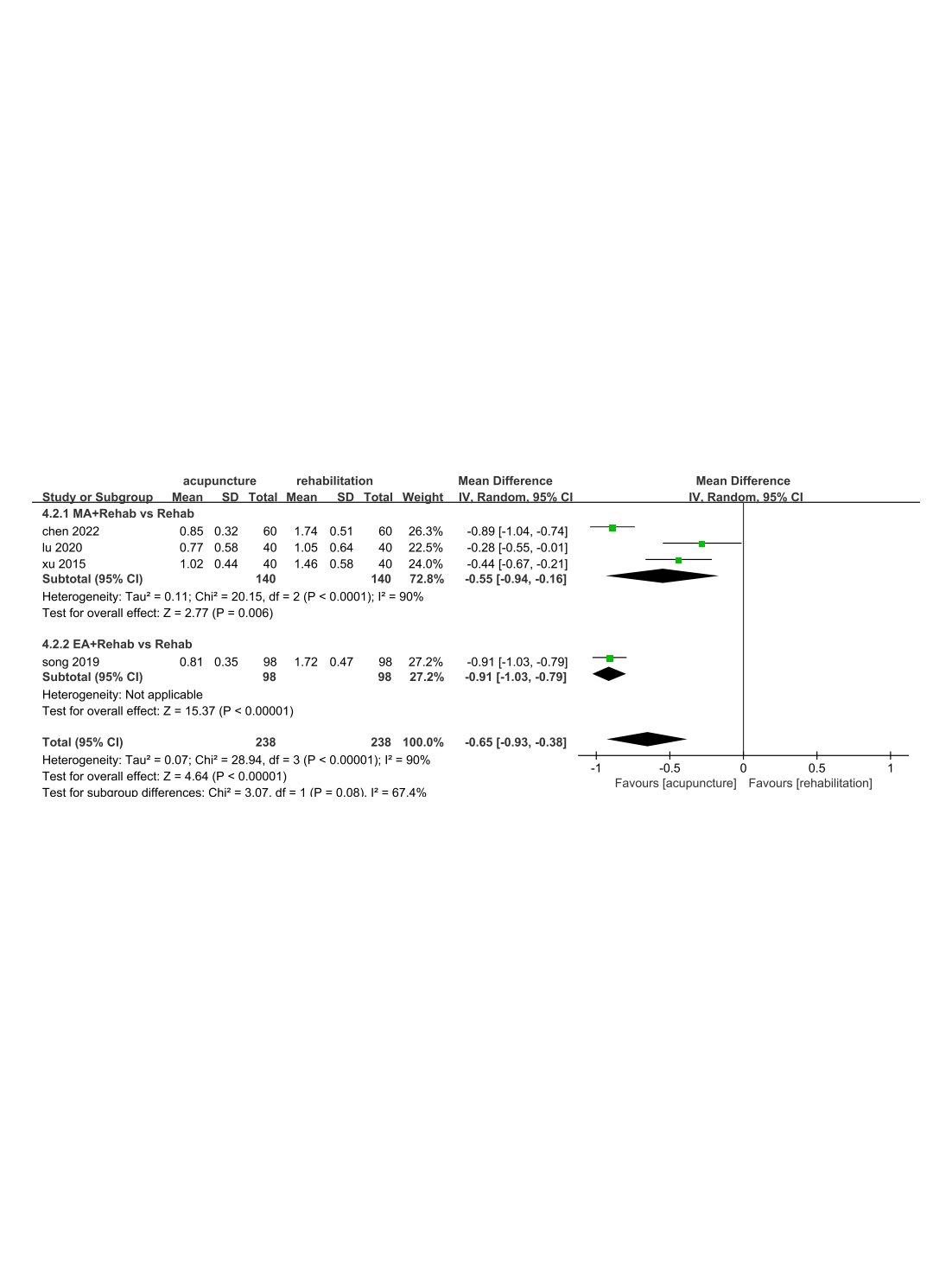

Supplement: SUPPLEMENTARY FIGURE 1 — Forest plot of acupuncture treatment combined with Rehab vs. Rehab on Edema. [file Data_Sheet_1.zip › Figure/Supplementary Figure 2.tif]

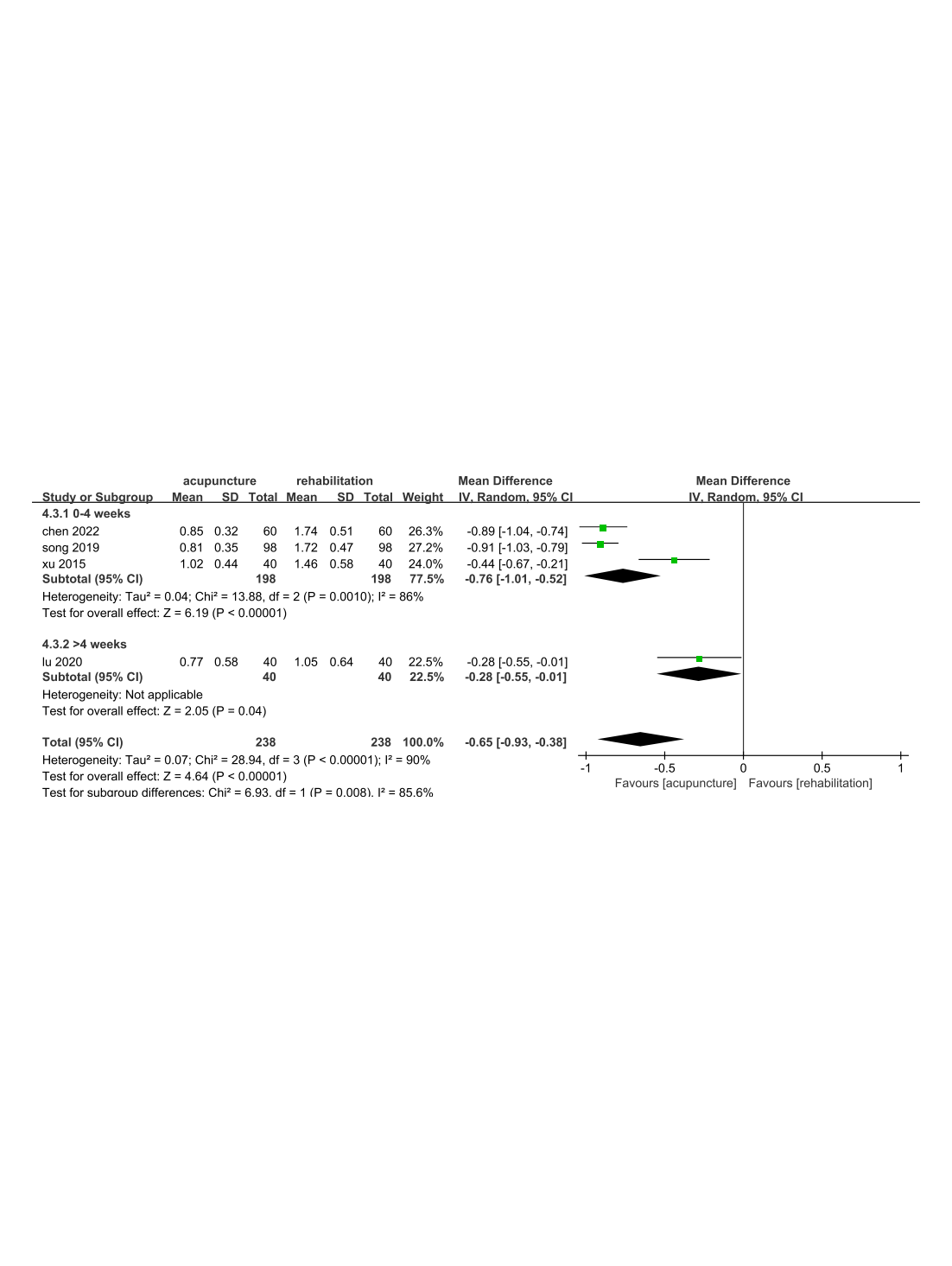

Supplement: SUPPLEMENTARY FIGURE 1 — Forest plot of acupuncture treatment combined with Rehab vs. Rehab on Edema. [file Data_Sheet_1.zip › Figure/Supplementary Figure 3.tif]

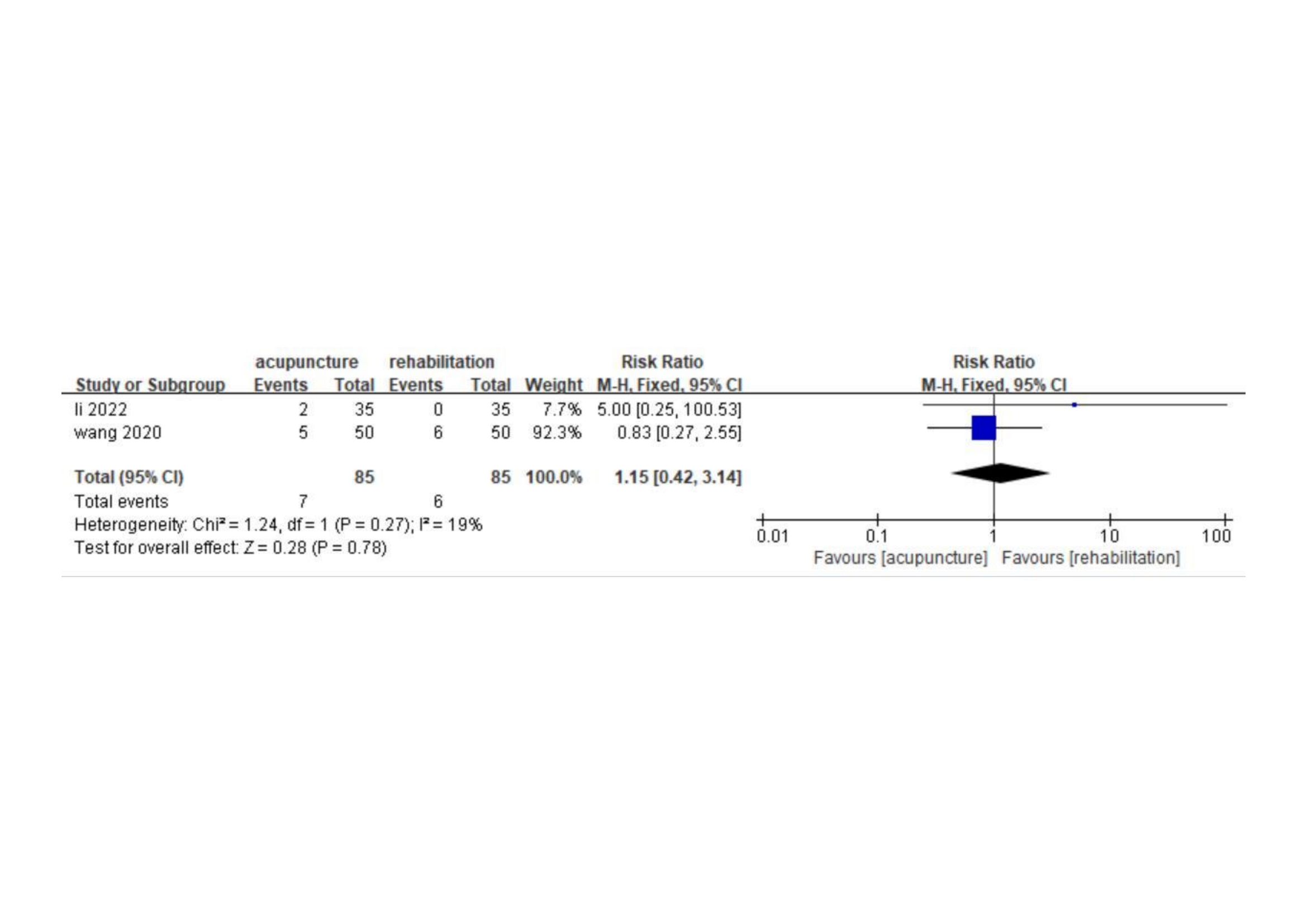

Supplement: SUPPLEMENTARY FIGURE 1 — Forest plot of acupuncture treatment combined with Rehab vs. Rehab on Edema. [file Data_Sheet_1.zip › Figure/Supplementary Figure 4.tif]

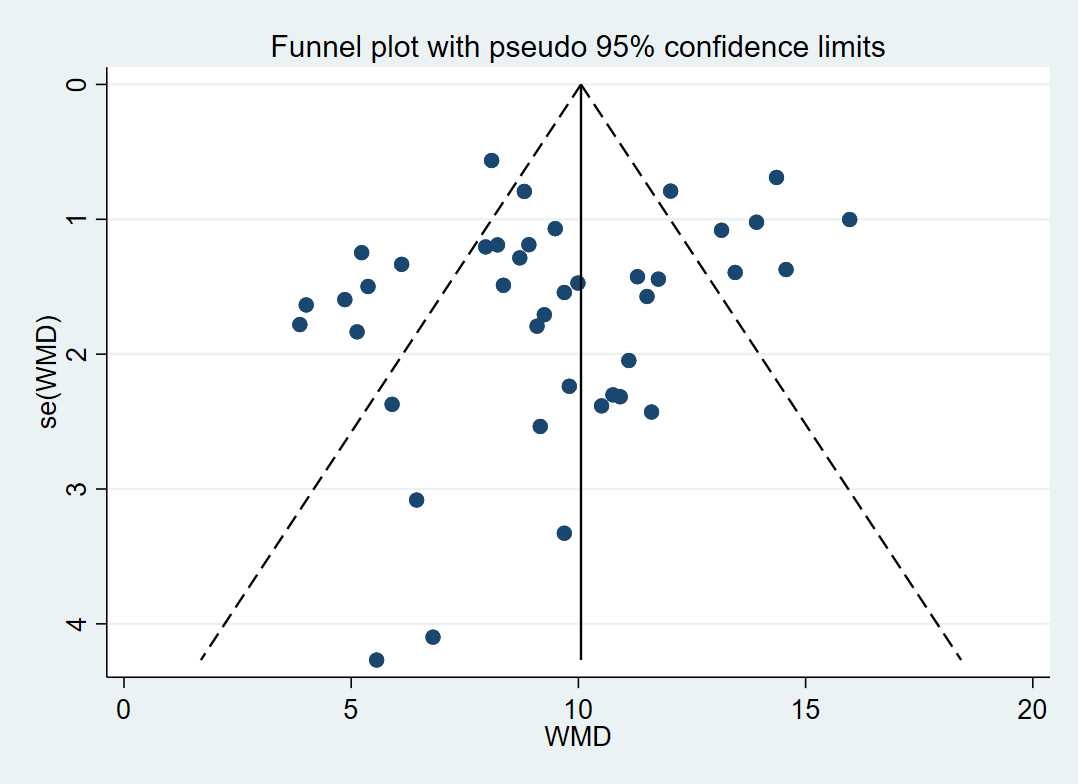

Supplement: SUPPLEMENTARY FIGURE 1 — Forest plot of acupuncture treatment combined with Rehab vs. Rehab on Edema. [file Data_Sheet_1.zip › Figure/Supplementary Figure 5.tif]

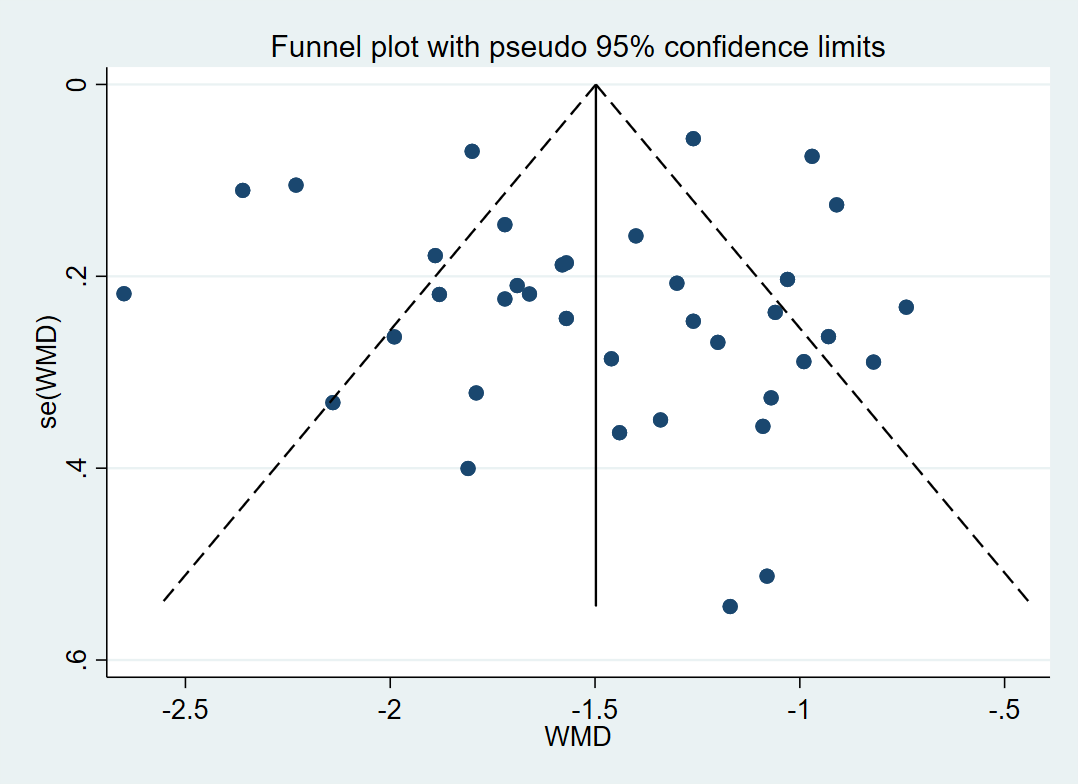

Supplement: SUPPLEMENTARY FIGURE 1 — Forest plot of acupuncture treatment combined with Rehab vs. Rehab on Edema. [file Data_Sheet_1.zip › Figure/Supplementary Figure 6.tif]

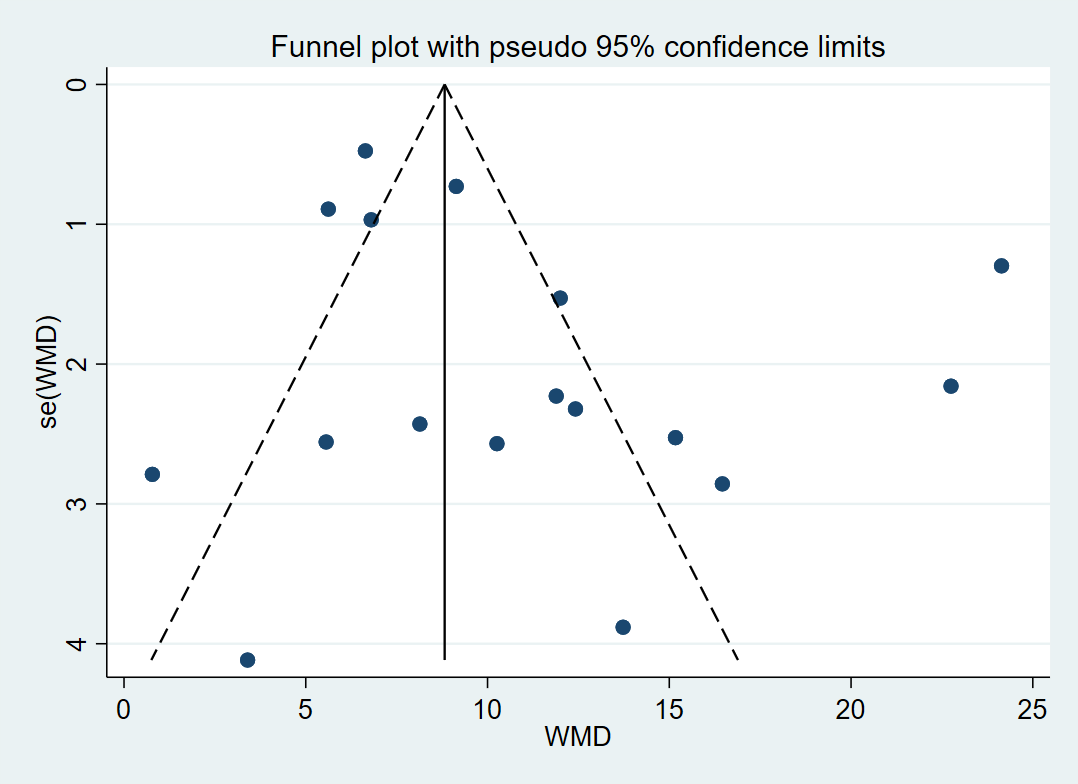

Supplement: SUPPLEMENTARY FIGURE 1 — Forest plot of acupuncture treatment combined with Rehab vs. Rehab on Edema. [file Data_Sheet_1.zip › Figure/Supplementary Figure 7.tif]
